# Deep Eutectic Solvents Formed by Complex Hydrides: A New Class of Hydrogen‐Rich Liquid

**DOI:** 10.1002/adma.202502566

**Published:** 2025-06-22

**Authors:** Loris Lombardo, Taichi Nishiguchi, Thi Ha My Pham, Andreas Züttel, Satoshi Horike

**Affiliations:** ^1^ Institute of Chemical Sciences and Engineering Basic Science Faculty École polytechnique fédérale de Lausanne (EPFL) Valais/Wallis Energypolis, Rue de l'Industrie 17 Sion 1951 Switzerland; ^2^ EMPA Materials Science and Technology Dübendorf 8600 Switzerland; ^3^ Department of Chemistry Graduate School of Science Kyoto University Kitashirakawa‐Oiwakecho, Sakyo‐ku Kyoto 606–8502 Japan; ^4^ Institute for Integrated Cell‐Material Sciences Institute for Advanced Study Kyoto University Yoshida‐Honmachi, Sakyo‐ku Kyoto 606–8501 Japan; ^5^ Department of Materials Science and Engineering School of Molecular Science and Engineering Vidyasirimedhi Institute of Science and Technology Rayong 21210 Thailand

**Keywords:** ammonia borane, borohydride, deep eutectic solvents, liquid hydrogen carrier

## Abstract

Deep eutectic solvents (DESs) are novel mixtures that exhibit a significant depression in melting points compared to their individual components. This work finds that combining tetrabutylammonium borohydride (TBABH) with ammonia borane (AB) yields new, stable, hydrogen‐rich liquids under ambient conditions, with a glass transition as low as −50 °C. Liquid mixtures containing up to 6.9 wt% hydrogen can be easily obtained through physical grinding. The strong interaction between the BH_4_^−^ anion of TBABH and AB coupled with the vibration dynamics of the alkyl chains accounts for the sharp decrease in melting point. The eutectic point is identified at a TBABH‐AB molar ratio of 1–2. Increasing the AB ratio further reduces the glass transition temperature but also induces a cold crystallization phenomenon. These mixtures can release hydrogen at temperatures as low as 60 °C, making them promising candidates for hydrogen storage. This represents the first example of a hydride‐based DES, advancing research on complex hydrides and opening the door to the discovery of new hydrogen‐rich liquids for various applications.

## Graphical abstract

This work discovers a new type of deep eutectic solvent composed of ammonia borane (NH_3_BH_3_, AB) and tetrabutylammonium borohydride (TBABH). The new liquids possess a hydrogen content higher than 5 wt%, thus representing promising high‐energy liquid hydrogen carriers. The strong hydrogen bonding between AB and TBABH explains the depletion of the melting point, with the eutectic point sitting at a molar ratio of 1–2 TBABH‐AB.

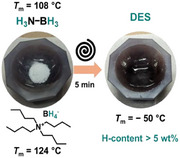

## 1 Introduction

Complex hydrides, such as alanates and borohydrides, alongside ammonia borane (NH_3_BH_3_, AB) are promising solid‐state materials for energy storage.^[^ ^1^, ^2^, ^3^ ^]^ These compounds have been extensively studied as hydrogen storage materials due to their high hydrogen content (AB: 19.6 wt%) and as potential CO_2_‐reducing agents.^[^ ^4^, ^5^ ^]^ AB is a crystalline solid at room temperature with a density of 0.78 g cm^−3^ at 25 °C and decomposes upon heating at ≈100 °C. However, AB suffers from poor H_2_ release kinetics and the simultaneous emission of by‐products such as ammonia and diborane.^[^ ^6^ ^]^ Several approaches have been tested to enhance H_2_ release and improve the purity of AB, with nanoconfinement and additives showing particularly encouraging results.^[^ ^7^, ^8^, ^9^, ^10^, ^11^ ^]^ Combining AB with other complex hydrides or metal hydrides is especially appealing, as the hydrogen content of the mixtures remains high.^[^ ^12^, ^13^ ^]^ The mixtures often decompose at lower temperatures and with reduced amounts of by‐products thanks to new H_2_ release pathways. A less studied method is to mix organic hydrides or ionic liquids (ILs) with conventional complex hydrides to modify their melting behavior.^[^ ^14^, ^15^, ^16^ ^]^ Some organic borohydrides, such as methylguanidinium borohydride,^[^ ^17^ ^]^ 1‐allyl‐3‐methylimidazolium borohydride,^[^ ^18^ ^]^ and 1‐butyl‐3‐methylimidazolium borohydride,^[^ ^19^ ^]^ have also been found to be liquid at room temperature. Liquefaction of complex hydrides under ambient conditions would represent a significant advancement. Stable liquid hydrides could have multiple applications, including liquid hydrogen carriers, liquid reducing agents, and hypergolic propellants. To date, the liquefaction of ammonia borane (*T*_m_ = 104 °C) has only been reported by Fan et al., who achieved it through ammonia absorption at 0 °C.^[^ ^20^ ^]^ This process is reversible, rendering the liquid AB‐NH_3_ complex unstable at room temperature. Dissolving AB in ILs also creates a liquid fuel with faster H_2_ release, but the maximum H_2_ content is limited to 5 wt% due to the solubility constraints.^[^ ^14^, ^21^ ^]^

Deep eutectic solvents (DESs) are an emerging class of liquids formed by mixing a hydrogen bond donor (HBD) with a hydrogen bond acceptor (HBA).^[^ ^22^, ^23^ ^]^ The strong intermolecular interactions in DESs produce mixtures with unusually low melting points (*T*_m_) relative to their components, enabling the formation of new liquids with properties similar to those of ILs. DESs have been used as green solvents in applications such as synthesis, gas capture and separation, catalysis, metal processing, and more.^[^ ^24^, ^25^, ^26^, ^27^ ^]^ The most common DESs are composed of a quaternary ammonium salt and an organic HBD.^[^ ^28^ ^]^ Despite the number of known DESs, no report currently describes DESs containing hydride moieties.

Inspired by DESs and in the search for new additives for AB destabilization, we report for the first time the formation of deep eutectic mixtures between AB and tetrabutylammonium borohydride (TBABH). TBABH was chosen as HBD due to its commercial availability, the presence of BH_4_^−^ anion to maximize interactions with AB and hydrogen content, and the widespread use of tetrabutylammonium cations in DESs. Molar ratios of AB and TBABH ranging from 1–1.5 to 1–3.5 form stable liquids at room temperature with a glass transition temperature ≈−50 °C. When the molar equivalent of AB exceeds 2.5, cold crystallization is observed. We describe these mixtures' physical and chemical properties using various spectroscopic techniques. Additionally, in situ Raman spectroscopy and density functional theory (DFT) calculations were performed to understand the interactions within this new class of hydrogen‐rich liquids.

## 2 Results and Discussion

### 2.1 Melting Behavior of the Hydridic Mixture

**Figure 1:**
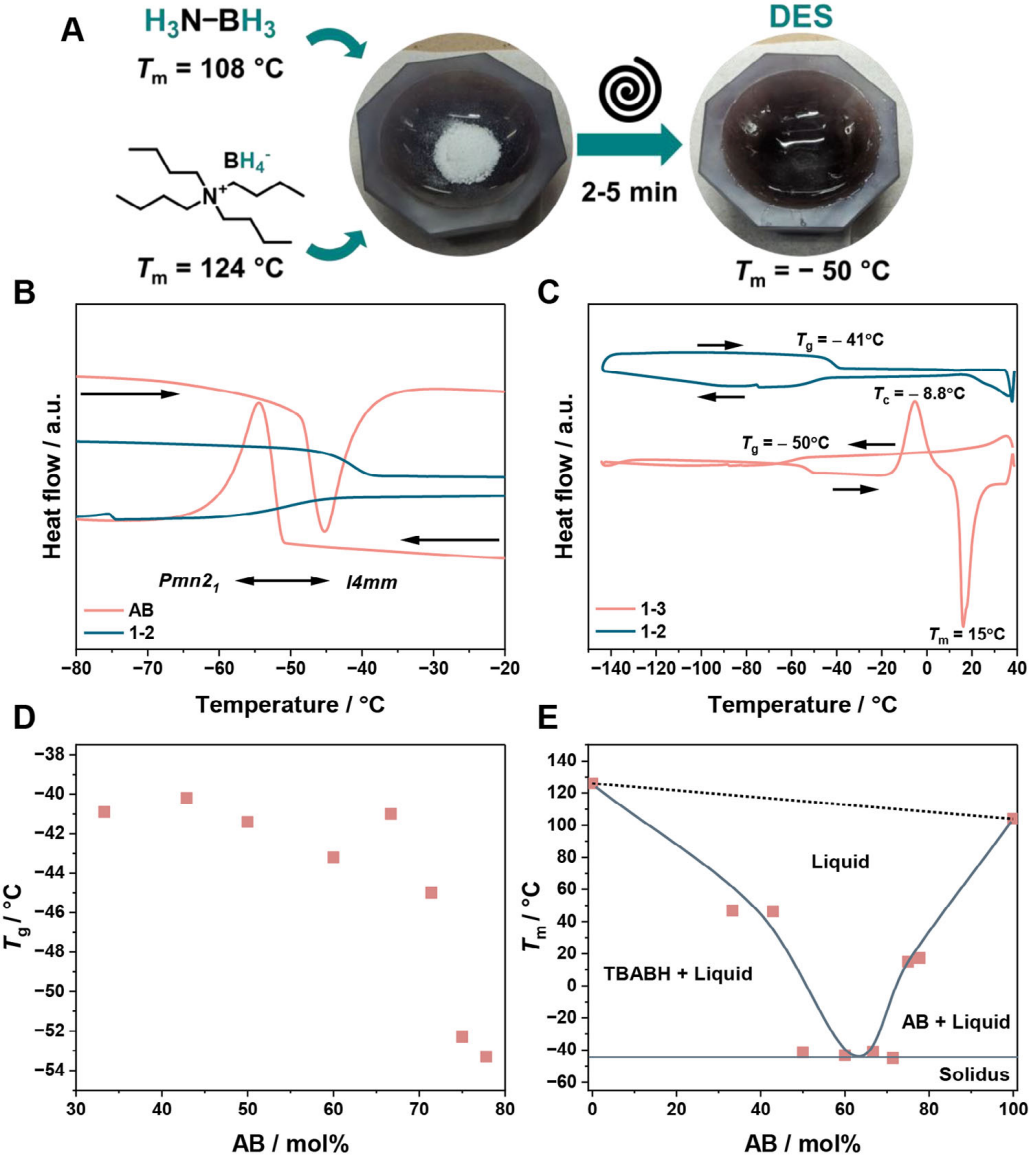
A) Schematic representation of the DES preparation by mixing AB and TBABH, leading to a translucide liquid at ambient conditions. DSC profiles comparison between B) AB and TBABH‐AB 1–2 mixture, and C) TBABH‐AB 1–2 and 1–3 mixtures (second cycle, 10 K min^−1^). D) Evolution of the *T*_g_ as a function of the molar amount of AB. E) Phase diagram of TBABH and AB mixtures. The solidus points refer to the *T*_g_. The values were determined by DSC measurements from the second heating cycle (10 K min^−1^).

We began by identifying the eutectic point between TBABH and AB through physical mixing under Ar in various ratios. Room temperature liquid‐gel mixtures formed when the molar amount of AB was between 50% and 80%. Specifically, TBABH‐AB molar ratios between 1–2 and 1–3 produced especially clear translucent liquids (**Figures** **1**A, and S1, Supporting Information). Powder X‐ray diffraction (PXRD) of the 1–2 mixture confirms the amorphous nature of the liquid (Figure S2, Supporting Information). By summing the hydrogen content of AB and BH_4_^−^, we determined the theoretical hydrogen content of the new DESs, which ranges from 4.3 to 6.9 wt%, surpassing the 2025 DOE target (Figure S3, Supporting Information).^[^ ^29^ ^]^ It is important to point out that no room temperature liquid was formed with smaller alkyl chains (ethyl and methyl), suggesting the significance of molecular motion and free volume, as described in traditional DESs (Figure S4, Supporting Information).^[^ ^30^, ^31^ ^]^ For instance, a longer alkyl chain expands non‐polar domains and creates larger void spaces between cations, disturbing dense packing.

Differential scanning calorimetry (DSC) of pure AB shows the typical tetragonal‐to‐orthorhombic crystal transition at −54 °C, while the 1–2 mixture of TBABH and AB only shows a glass transition (*T*_g_) at −41 °C (Figure 1B).^[^ ^32^, ^33^, ^34^ ^]^ Other liquid‐forming ratios also display a glass transition between −40 and −53 °C (Figure 1C and Figure S5, Supporting Information). The *T*_g_ decreases with the increase in AB content (Figure 1D). *T*_g_ can be interpreted as a change in dynamics; previous studies have linked the glass transition temperature with molecular mobility, where adding HBD reduces the viscosity.^[^ ^35^ ^]^ To assess if the observed decrease in *T*_g_ correlates with AB content, viscosity measurements were conducted for the 1–2 and 1–3 mixtures. While their viscosities from room temperature to −20 °C are similar, the 1–2 sample experiences a sharp increase in viscosity at lower temperatures (Figure S6, Supporting Information). At −40 °C, close to the glass transition, the viscosity of the 1–2 mixture is ≈1000 times higher than that of the 1–3 mixture, confirming the superior molecular mobility with increasing HBD content. We also noticed that the DESs behave like non‐Newtonian liquids with a shear‐thinning effect (Figure S7, Supporting Information), which was already observed in other eutectic mixtures.^[^ ^36^ ^]^ This effect arises from the rearrangement of TBABH and AB molecules (alignment, disentangle, and H‐bonding breaking). The DSC analysis also reveals a crystallization peak (*T*_c_) when the molar ratio of AB exceeds 2.5, occurring at −8.8 °C for the 1–3 mixture and −16.4 °C for the 1–3.5 mixture (Figure 1C and Figure S4, Supporting Information). If AB is present in excess, intermolecular interactions among AB molecules can lead to partial re‐crystallization of AB. Crystallization only occurs when the sample is cooled below its *T*_g_ (Figure S8, Supporting Information), thus classified as cold crystallization. Cold crystallization happens when a material stores excess thermal energy during cooling.^[^ ^37^, ^38^ ^]^ Compounds exhibiting cold crystallization often contain flexible moieties and a non‐uniform structure, which helps maintain a high degree of molecular motion. The H‐bonding between TBABH and AB, coupled with the flexible alkyl chain, likely contributes to cold crystallization. Following cold crystallization, an endothermic peak appears in the DSC corresponding to the *T*_m_ at 15 and 17.4 °C for 1–3 and 1–3.5 (Figure 1C and Figure S5, Supporting Information). When the molar ratio of AB is less than 1, the mixtures are solid at room temperature with a *T*_m_ of ≈45 °C (**Table** **1**, Figure S9, Supporting Information). A tentative phase diagram is shown in Figure 1E where we consider the *T*_m_ after cold crystallization for the 1–3 and 1–3.5 ratio. The eutectic point occurs at ≈70 mol% of AB.

**Table 1:** *T*_m_, *T*_g_, and *T*_c_ of TBABH‐AB with different molar ratios.

TBABH‐AB mol ratio	T_m_ / °C	T_g_ / °C	T_c_ / °C
1‐0	129	/	/
2‐1	46.8	−40.9	1
4‐3	46.4	−40.2	2.2
1‐1	/	−41.4	/
1‐1.5	/	−43.2	/
1‐2	/	−41	/
1‐2.5	/	−45	/
1‐3	15	−52.3	−8.8
1‐3.5	17.4	−53.3	−16.4
0‐1	104	/	/

To confirm that the TBABH‐AB are DES and not simply eutectic mixtures, we determine the ideal eutectic temperature for the 1–2 mixture using the Schröder‐van‐Laar equation (see details in Supporting Information). As the melting enthalpy of AB cannot be determined due to decomposition upon melting, we assessed the melting enthalpy of TBABH (Figure S10, Supporting Information). The *ΔH_fus_* was found to be 15.8 kJ·mol^−1^, close to TBABr (*ΔH_fus_* = 16.5 kJ·mol^−1^).^[^ ^39^ ^]^ The theoretical eutectic temperature for a molar ratio of 1/3 (TBABH‐AB 1–2) was calculated at 325.2 K (52 °C), 100 °C higher than the observed *T*_g_, confirming the deep eutectic behavior of the system (Figure S11, Supporting Information). Although 1–3 displays cold crystallization, it can be prevented by maintaining the mixture above −50 °C, making it a promising liquid hydrogen carrier with a hydrogen content of 6.35 wt%. The refractive indices with regard to the sodium D line (*n*_D_) of the 1–2 and 1–3 mixtures are close at 1.4771 and 1.4753, respectively, in the same range as other DESs (Table S1, Supporting Information).^[^ ^40^ ^]^ However, the density was measured at 0.766 and 0.747 g·cm^−3^ for 1–2 and 1−3, respectively. These values are among the lowest in DES due to the low molecular weight and density of AB compared to previously reported DESs.^[^ ^41^ ^]^ It indicates the presence of free volume in the DES since the density of AB is slightly higher (0.78 g·cm^−3^).

### 2.2 Interactions Between AB and TBABH

**Figure 2:**
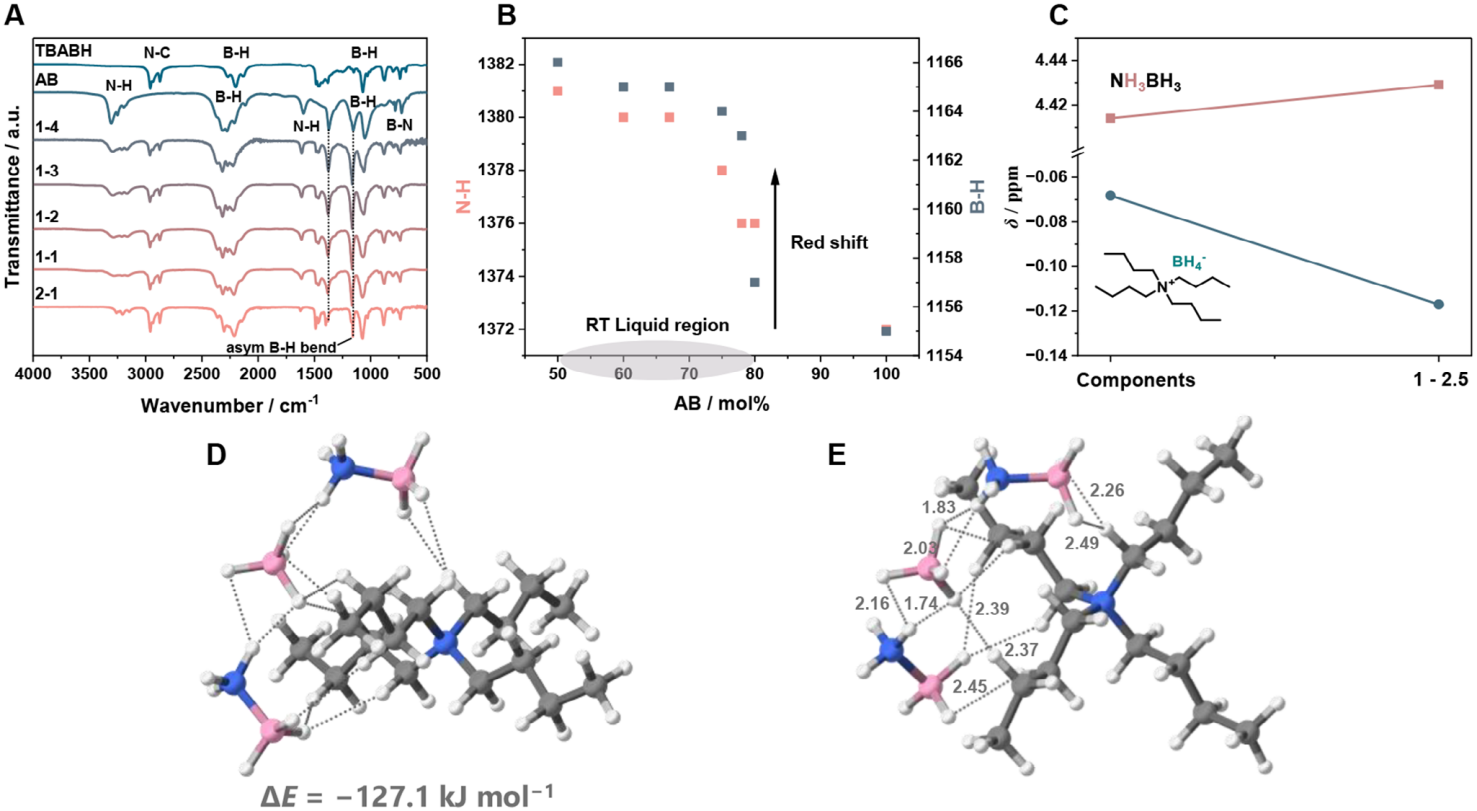
A) FT‐IR spectra of TBABH, AB, and their physical mixtures with different molar ratios. B) The evolution of AB symmetric N−H and asymmetric B−H bend peak positions upon mixing with TBABH. C) Comparison of ^1^H NMR chemical shifts in the TBABH‐AB 1–2.5 with its constituents. D) Gas phase optimized molecular structure of 1–2 TBABH‐AB DES using B3LYP/6‐311++G(d,p). E) Intermolecular H−H bonding distance between AB and TBABH. H, white; C, grey; N, blue; B, pink.

Nuclear magnetic resonance (NMR) and Fourier‐transform infrared (FT‐IR) spectroscopy confirm the formation of hydrogen bonds between TBABH and AB (**Figure** **2**). The N−H stretching signals between 3248 and 3310 cm^−1^ in the DESs, originating from NH_3_BH_3_, broaden relative to pristine AB, indicating changes in the chemical environment due to H‐bonding (Figure 2A). Meanwhile, the typical boron−hydrogen (B−H) stretching signals of AB and TBABH ≈2225 cm^−1^ overlap, validating the presence of both BH_3_ and BH_4_^−^. A clear red shift is also observed in the symmetric N−H bend at 1372 cm^−1^ and B−H bend at 1155 cm^−1^ of AB (Figure 2B). Shifts to lower frequencies are common in DESs, resulting from changes in hydrogen bonding as pristine AB transitions to the DES form.^[^ ^42^ ^] 11^B and ^1^H NMR confirm the presence of BH_3_ and BH_4_^−^ species without decomposition (Figure S12, Supporting Information). The ^1^H NMR shift of the BH_4_^−^ anion also suggests its involvement in the H‐bonding (Figure 2C).

To further comprehend the local interaction geometries between TBABH and AB, DFT geometry optimization using B3LYP/6‐311++G(d,p) level of theory was performed (Figure 2D,E). Two hydride (H^δ−^) atoms of the BH_4_^−^ anion interact with a protonic H atom of AB, forming strong H^δ−^··· ^δ+^H bonds with an intermolecular distance ranging from 1.74 to 2.16 Å. A weaker hydrogen bond also occurs between the alkyl chain H atoms of TBABH and BH_3_ group of AB, with intermolecular distances between 2.26 and 2.49 Å. The overall energy of the system composed of one TBABH and two AB molecules is favourable (Δ*E* = −127.1 kJ·mol^−1^). Simulated IR spectra for TBABH, AB, and the 1–2 DES display frequencies similar to those in the experimental FT‐IR spectra (Figure S13, Supporting Information), with a red shift in the DES for the peaks between 1000 and 1350 cm^−1^. The peak position differences arise from the lack of large‐scale interaction present in the liquid, as the DFT modeling is performed in a vacuum. An alternative optimal geometry was found, in which only one AB interacts with BH_4_^−^, though it has a slightly higher overall energy (Δ*E* = −74.6 kJ mol^−1^, Figure S14, Supporting Information).

Based on theoretical and experimental data, we can conclude that the melting depletion originates from two main factors: the rich H‐bonding between AB and TBABH and the dynamics of the alkyl chain. The various possible H‐bonds diminish the bonding strength and allow the system to flow at a wide range of temperatures.^[^ ^43^ ^]^ In addition, longer chains expand non‐polar domains and create larger void spaces (or free volume) between cations, disturbing dense packing, thus preventing crystallization. At low temperatures, the free volume arising from the vibrational motion diminishes until a critical point at which the sample turns into a glass.^[^ ^44^ ^]^

**Figure 3:**
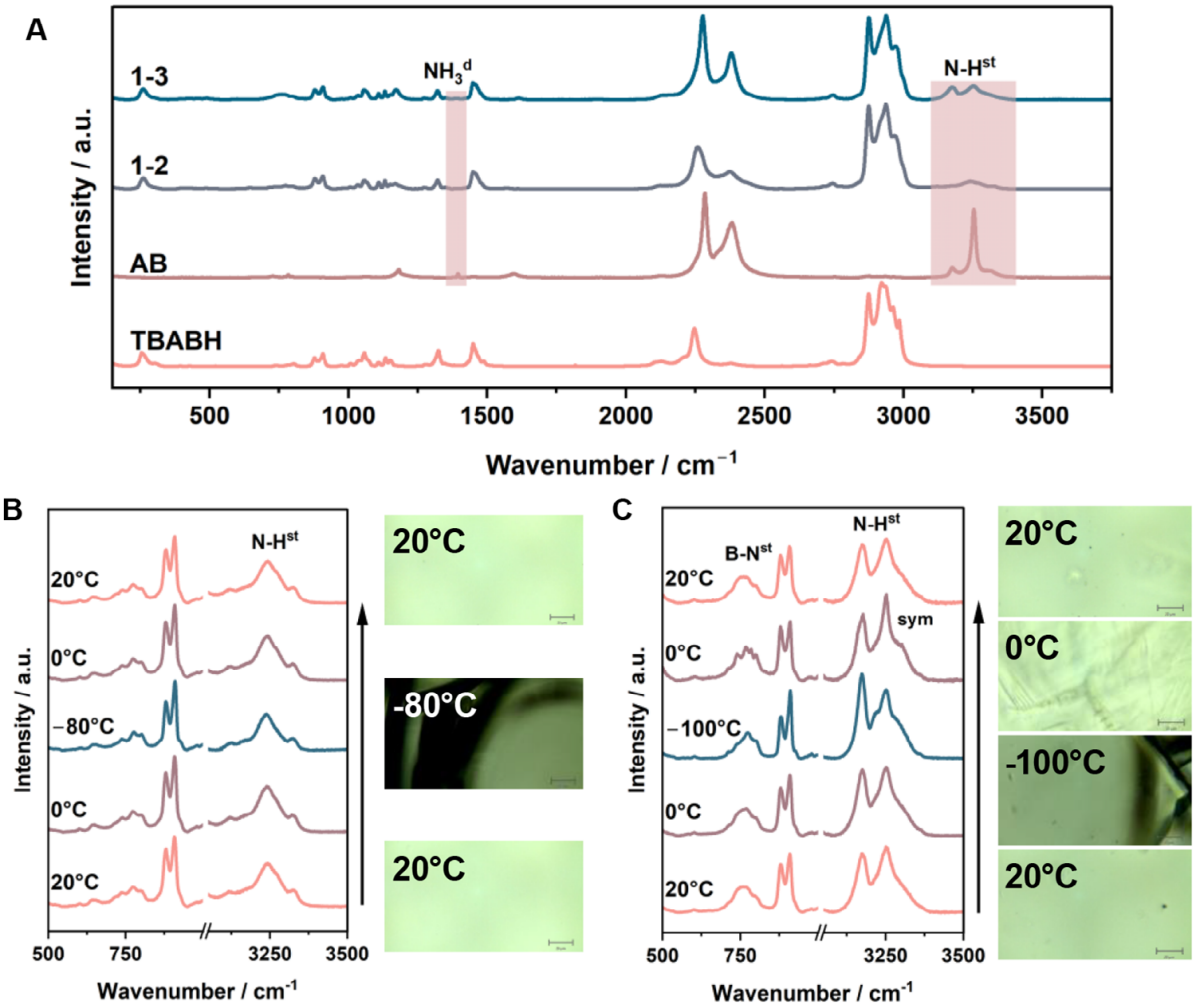
A) Raman spectra of TBABH, AB, 1–2, and 1–3 TBABH‐AB mixtures. B) In situ Raman spectra of TBABH‐AB 1–2 showing the evolution of AB N−H stretching mode and sample appearance as a function of temperature. C) In situ Raman spectra of TBABH‐AB 1–3 showing the evolution of AB N−H and B−N stretching mode, and sample appearance as a function of temperature.

To better understand the interaction dynamics during the glass transition and cold crystallization, we performed in situ Raman spectroscopy on the 1–2 and 1–3 mixtures (**Figure** **3**). The spectra of these mixtures display the combined features of the references AB and TBABH, with AB peaks being more prominent in the 1–3 mixture due to its excess (B−N stretching at 785 cm^−1^, B−H stretching at 2280 and 2375 cm^−1^, N−H stretching at 3174, 3252, and 3322 cm^−1^).^[^ ^45^, ^46^, ^47^, ^48^ ^]^ No new peaks were detected in either mixture, confirming the molecular integrity during DES formation (Figure 3A). While no significant changes were observed in the TBABH peaks or the B−N and B−H stretching peaks of AB, we noticed that the peak corresponding to the N−H stretching mode of AB in the mixture was broadened. This broadening is attributed to the loss of crystallinity of AB in the mixture compared to the crystalline bulk reference, as previously confirmed by PXRD. In the 1–2 DES, apart from the three main peaks at 3120, 3240, and 3327 cm^−1^, two additional shoulders appear at 3170 and 3273 cm^−1^, suggesting that this broad peak can be deconvoluted into five peaks for a more in‐depth analysis of different N−H stretching modes in the mixture (Figure S15, Supporting Information). This deconvolution is consistent with the Raman peak simulations obtained through DFT for the 1–2 mixture with the configuration shown in Figure 2D (Table S2, Supporting Information). The asymmetric stretching of NH_3_ is the dominant N−H stretching mode in the eutectic solution, showcasing the strong interactions arising from hydrogen bonding between NH_3_ of AB and BH_4_^−^ of TBABH.

The cryogenic properties of the DES were further investigated using in situ Raman spectroscopy during cooling from room temperature to −100 °C, followed by heating back to room temperature (Figures S16 and S17, Supporting Information). For the 1–2 mixture, which is the eutectic point, the Raman spectra remained unchanged throughout the entire cooling process (Figure 3B), even though the microscopic images revealed cracks forming at −80 °C due to shrinking below the glass transition temperature. These cracks explain the minor response observed in DSC ≈−80 °C (Figure 1C). Similarly, no spectral change was observed during the heating process, despite the melting observed ≈−40 °C in the microscopic images (Supporting Video S1, Supporting Information). The deconvoluted N−H stretching peaks remain stable over the cooling‐heating cycle, confirming a similar structure between the glass and liquid phases.

In the case of the 1–3 mixture, no significant changes in the Raman spectra were observed during cooling from room temperature to –60 °C (Figure S17, Supporting Information). However, some peaks became sharper as the glass formed such as the N−H stretching peak of AB and the aliphatic C−C at 910 cm^−1^ from TBABH (Figure 3C). Raman peak width is influenced by the molecular structure and intermolecular interactions. Going from liquid to glass reduces the disorder within the sample and can change the intermolecular interaction between excess AB and TBABH.^[^ ^49^ ^]^ The appearance of a new peak shoulder at 3220 cm^−1^ in the N−H stretching region of AB below −60 °C is attributed to the reduction in dynamic disorder. As the material transitions from a liquid to a glassy state, reduced dynamic averaging and stabilization of molecular conformations lead to more distinct vibrational modes, resulting in peak splitting.^[^ ^50^ ^]^ During the heating process, the sample melts again before crystallizing. Once cold crystallization was completed at 0 °C, a stronger presence of the N−H band at 3252 cm^−1^ was noticed (Figure 3C). This peak can be attributed to the symmetric vibration mode in the newly formed crystal phase, which is consistent with the strong peak intensity from the symmetric stretching observed in the bulk AB reference. Additionally, the NBH rocking and B−N stretching peaks in the range of 740 to 800 cm^−1^ sharpened and became more defined as crystals formed.^[^ ^48^ ^]^ The formation of crystal dendrites was also observed at this temperature (Supplementary Video S2), before melting occurred at room temperature. As melting begins, the mixture returns to a liquid phase, in which the symmetric N−H stretching mode is less prominent.

### 2.3 Thermal Stability and Decomposition

**Figure 4:**
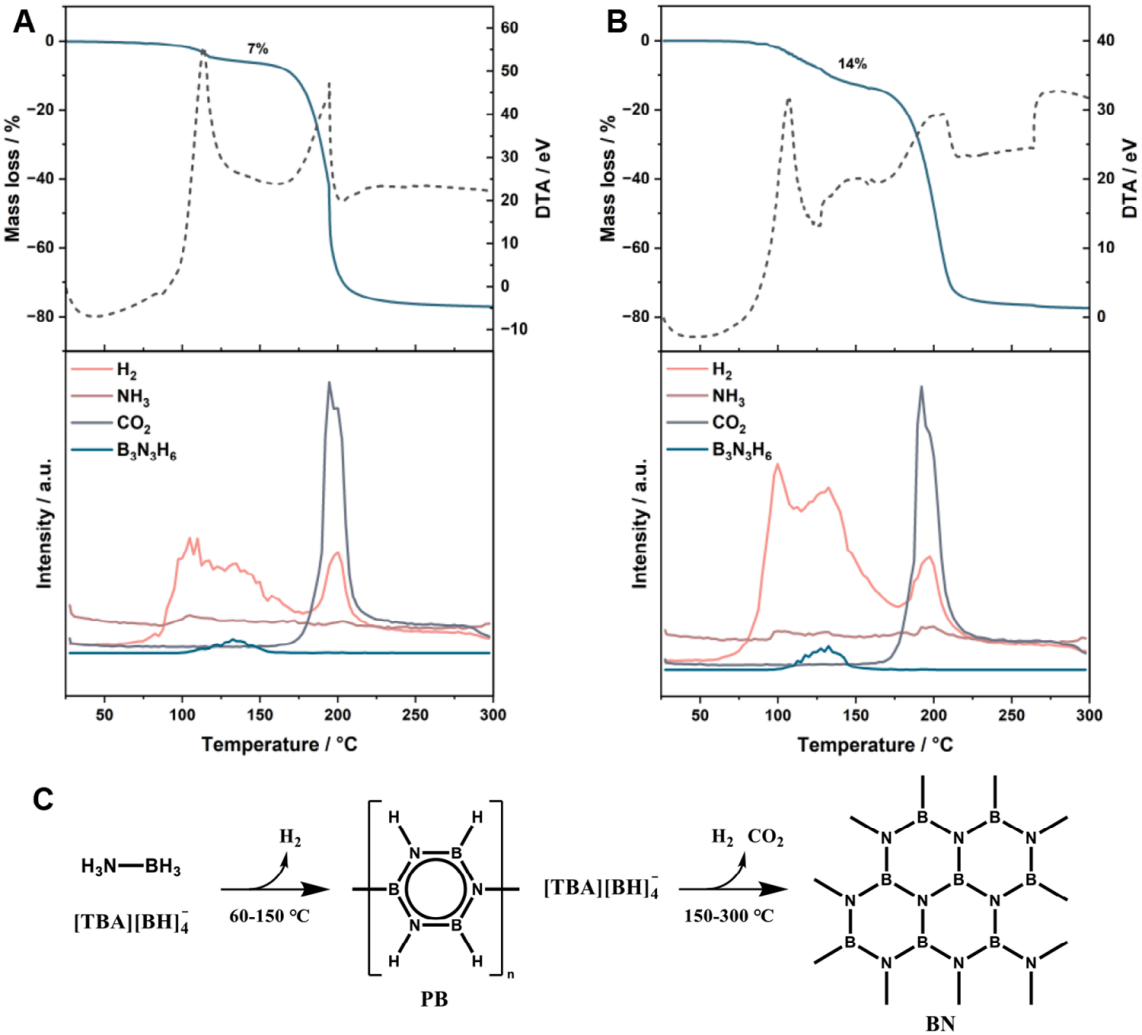
TG‐DTA and the corresponding MS signals of A) 1–2 TBABH‐AB and B) 1–3 TBABH‐AB DES. C) Proposed decomposition mechanism of the DES.

Next, the stability of the new hydride‐based DES was evaluated through thermal analysis. No decomposition was noticed by FT‐IR for the 1–2 and 1–3 mixtures stored under Ar for over 1 month (Figure S18, Supporting Information). However, H_2_ is slowly released in air due to the hydrolysis of AB and TBABH to B(OH)_3_ in the presence of moisture (Figures S19 and S20, Supporting Information). The DES keeps the hygroscopic nature of TBABH and AB and should be stored in a dry environment. Thermogravimetric and differential thermal analysis (TG‐DTA) and thermogravimetric analysis and mass spectroscopy (TG‐MS) measurements of TBABH‐AB 1–2 and 1–3 mixtures are shown in **Figure** **4**. Decomposition of the 1–2 DES occurs in two stages, with mass losses of 7% and 70% at 80 and 165 °C, respectively. A similar trend is observed for the 1–3 mixture, though it exhibits a 14% mass loss in the first step. The difference in mass loss comes from the difference in the AB ratio. The gases that evolved during the first step are primarily hydrogen, with trace amounts of ammonia and borazine (B_3_N_3_H_6_). H_2_ release begins at ≈60 °C, followed by NH_3_ at ≈90 °C, and borazine at ≈100 °C. These species are characteristic of AB thermal decomposition, suggesting that only AB decomposes during the first stage.^[^ ^6^, ^51^, ^52^ ^]^ This aligns with the larger amount of H_2_ evolved in the 1–3 sample. The dehydrogenation temperature is lower than that of pure AB due to the borohydride‐induced destabilization of AB and the amorphous nature of the sample.^[^ ^12^, ^53^ ^]^ The activation energy of AB decomposition was estimated by Kissinger analysis at 117.8 kJ·mol^−1^ (Figure S21, Supporting Information), lower than pure AB (>160 kJ·mol^−1^).^[^ ^54^, ^55^ ^]^ Our *E_a_* is close to what was reported for AB dissolved in diglyme (113 kJ·mol^−1^).^[^ ^6^ ^] 11^B NMR of 1–2 sample measured after heat treatment to 100 and 150 °C confirms that only AB decomposes below 150 °C, indicated by the diminishing BH_3_ peak centered at −23 ppm. In contrast, the borohydride peak at −35 ppm remains, confirming the stability of TBABH up to 150 °C (Figure S22, Supporting Information). FT‐IR signals of NH_3_ and BH_3_ in AB at 3250 and 2300 cm^−1^ also fade at 150 °C (Figure S23, Supporting Information). New peaks located at 3430, 2505, and 1380 cm^−1^ suggest the formation of polyborazylene (PB) post‐dehydrogenation.^[^ ^56^, ^57^ ^]^ During AB dehydrogenation, the sample undergoes a phase transition from liquid to paste to translucid solid (Figure S24, Supporting Information), attributed to PB formation. This phenomenon was also observed for AB dehydrogenation in ILs.^[^ ^14^ ^]^ It is important to notice the absence of diammoniate of diborane ([H_2_B(NH_3_)_2_][BH_4_]) and polyaminoborane, which are typically observed during thermolysis of AB in the solid state.^[^ ^58^, ^59^ ^]^

At temperatures above 150 °C, TBABH decomposes, releasing H_2_, NH_3_, and CO_2_ (Figure 4). After heat treatment at 300 °C, a red residue was obtained and identified as boron nitride (BN) based on FT‐IR data (Figure 4C and Figure S16, Supporting Information).^[^ ^51^, ^56^, ^60^ ^]^ BN formation at temperatures below 500 °C is unusual; the interaction of PB with TBABH may create new reaction paths for the formation of BN at 300 °C, thanks to H‐bonding.^[^ ^53^, ^61^ ^]^ The isothermal H_2_ release kinetics were followed by TG‐MS at temperatures of 85, 90, 95, and 100 °C (Figures S25 and S26, Supporting Information). At temperatures below 95 °C, only H_2_ gas was observed by MS, suggesting that isothermal decomposition can suppress the release of impurities (Figure S27, Supporting Information). In addition, the release rate observed is almost 10 times faster than comparable systems where AB is dissolved in an IL or DES (Table S3, Supporting Information). The 1–2 mixture has a dehydrogenation rate close to that ofcatalytic AB methanolysis.^[^ ^62^ ^]^ As only AB decomposes in the first stage, recycling TBABH by separating it from AB by‐products is envisageable. Another method to release pure H_2_ would be the hydrolysis reaction, as we noticed H_2_ evolution upon exposure to moisture. Further research on re‐cyclability and other applications must be done to find the best applications for these new hydrogen‐rich liquids.

## 3 Conclusion

We discovered a new type of deep eutectic mixture through simple physical mixing of two complex hydrides: ammonia borane (AB) and tetrabutylammonium borohydride (TBABH). Stable liquid‐gel mixtures were formed with AB molar ratios between 50% and 80%. These samples exhibit glass transition upon cooling below −40 °C, confirming the significant melting point depletion compared to the parent compounds (TBABH: *T*_m_ = 126 °C; AB: *T*_m_ = 104 °C). Cold crystallization was observed in samples with AB molar equivalent greater than 2.5 due to partial recrystallization of AB. The eutectic point was found at a TBABH‐AB molar ratio of 1–2. Spectroscopic data and DFT calculation suggest strong hydrogen bonding between the BH_4_^−^ anion of TBABH and the NH_3_ group of two AB molecules. The melting point depletion was attributed to the rich H‐bonding between AB and TBABH and the presence of free volume originating from the molecular motion of the alkyl chains, avoiding dense packing. In situ Raman spectroscopy confirmed the chemical stability of the 1–2 mixture upon cooling below the glass transition and heating back to room temperature. In contrast, cold crystallization in the 1–3 mixture led to noticeable changes in the AB Raman signals. Finally, a two‐step thermal decomposition process was identified for the DESs: AB decomposed in the first step, followed by TBABH in the second. Hydrogen release began at temperatures as low as 60 °C, making these new hydride DESs promising candidates for liquid hydrogen carriers. These findings have implications for complex hydride research, not only for hydrogen storage but also as an advancement over solid‐state hydrides.

## Conflict of Interest

The authors declare no conflict of interest.

## Supporting information



Supporting Information

Supplemental Video 1

Supplemental Video 2

## Data Availability

The data that support the findings of this study are available in the supplementary material of this article.
