# Supplementary material for: Deep Eutectic Solvents Formed by Complex Hydrides: A New Class of Hydrogen‐Rich Liquid
Source: Adv Mater. 2025 Jun 22;37(37):2502566. doi: 10.1002/adma.202502566 (PMC12447031; doi:10.1002/adma.202502566)
Supplement: Supplementary file 1 — Supporting Information [file ADMA-37-2502566-s001.docx]

Supporting Information for

**Deep eutectic solvents formed by complex hydrides: a new class of hydrogen rich liquid**

Loris Lombardo,^*a,b,c^ Taichi Nishiguchi,^c^ Thi Ha My Pham,^a,b^ Andreas Züttel,^a,b^ Satoshi Horike ^*c,d,e^

*^a^Institute of Chemical Sciences and Engineering, Basic Science Faculty, École polytechnique fédérale de Lausanne (EPFL) Valais/Wallis, Energypolis, Rue de l’Industrie 17, CH-1951 Sion, Switzerland*

*^b^EMPA Materials Science and Technology, 8600 Dübendorf, Switzerland*

*^c^Department of Chemistry, Graduate School of Science, Kyoto University, Kitashirakawa-Oiwakecho, Sakyo-ku, Kyoto 606-8502, Japan*

*^d^Institute for Integrated Cell-Material Sciences, Institute for Advanced Study, Kyoto University, Yoshida-Honmachi, Sakyo-ku, Kyoto 606-8501, Japan*

*^e^Department of Materials Science and Engineering, School of Molecular Science and Engineering, Vidyasirimedhi Institute of Science and Technology, Rayong 21210, Thailand*

Table of Contents

[Experimental part 4](#_Toc197619691)

[Preparation of the mixtures 5](#_Toc197619692)

[Characterization 5](#_Toc197619693)

[DFT calculation 5](#_Toc197619694)

[Determination of the ideal eutectic temperature 6](#_Toc197619695)

[Results 7](#_Toc197619696)

[Figure S1: Pictures of the as-prepared DESs made from TBABH and AB with different molar ratio (TBABH-AB). 7](#_Toc197619697)

[Figure S2: PXRD pattern of TBABH, AB, and TBABH-AB 1-2. 7](#_Toc197619698)

[Figure S3: Theoretical hydrogen content of the AB and TBABH mixtures forming liquids based on the mole equivalent of AB. 8](#_Toc197619699)

[Figure S4: DSC profile of A TMABH-AB 1-2 and B TEABH-AB 1-2 heated to 100 °C. The endothermic peaks observed are due to partial melting or phase change, but it was not possible to produce a clear liquid at temperature below the decomposition of AB. 8](#_Toc197619700)

[Figure S5: First and second cycles DSC profile of A TBABH-AB 1-1.5, B TBABH-AB 1-2.5, and C TBABH-AB 1-3.5 cooled down up to -140 °C at 10 K min^−1^. 9](#_Toc197619701)

[Figure S6: Complex viscosity of the 1-2 and 1-3 DESs as a function of temperature. Cooling rate 5 K min^−1^, angular frequency 10 rad s^−1^, 10](#_Toc197619702)

[Figure S7: Viscosity as a function of the shear rate for A 1-2 and B 1-3 over two runs. 10](#_Toc197619703)

[Figure S8: First and second cycles DSC profile of TBABH-AB 1-3 cooled down up to -50 °C at 10 K min^−1^. 11](#_Toc197619705)

[Figure S9: First and second cycles DSC profile of A TBABH-AB 1-0.75 and B TBABH-AB 2-1 cooled down to -140 °C and heated up to 65 °C at 10 K min^−1^. 11](#_Toc197619706)

[Figure S10: DSC curve of TBABH melting to determine the melting enthalpy. 12](#_Toc197619707)

[Figure S11: Estimation of the theoretical eutectic temperature from the Schröder-van-Laar equation. 12](#_Toc197619708)

[Table S1: 13](#_Toc197619709)

[Figure S12: A ^11^B and B ^1^H NMR of TBABH-AB 1-2 (solvent: DMSO-*d_6_*). 13](#_Toc197619710)

[Figure S13: Simulated FT-IR spectra of TBABH, AB, and TBABH-AB 1-2. 14](#_Toc197619711)

[Figure S14: Gas phase optimized molecular structure of 1-2 TBABH-AB DES with only one AB interacting with BH_4_^−^ using B3LYP/6-311++G(d,p). 14](#_Toc197619712)

[Figure S15: *In-*situ Raman spectra of the NH^st^ region and their deconvolution for TBABH-AB 1-2 DES at A −80 °C, B −60 °C, C −40 °C, D −20 °C, E 0 °C, and F 20 °C. 15](#_Toc197619713)

[Table S2: Calculated Raman peak position in the NH_3_ region based on the DFT model. 16](#_Toc197619714)

[Figure S16: *In-*situ Raman spectra of TBABH-AB 1-2 DES during A cooling to −80 °C and B heating back to 20 °C. Cooling/heating rate: 10 K min^−1^. 17](#_Toc197619715)

[Figure S17: *In-*situ Raman spectra of TBABH-AB 1-3 DES during A cooling to −100 °C and B heating back to 25 °C. Cooling/heating rate: 10 K min^−1^. 17](#_Toc197619716)

[Figure S18: Evolution of FT-IR spectra over time for A TBABH-AB 1-2 and B TBABH-AB 1-3. 18](#_Toc197619717)

[Figure S19: Evolution of FT-IR spectra over time for TBABH-AB 1-2 exposed to air (20 °C, RH 30%). 18](#_Toc197619718)

[Figure S20: ^11^B NMR of the 1-2 DES before and after 20h air exposure (solvent: DMSO-*d_6_*). 19](#_Toc197619719)

[Figure S21: Kissinger analysis on the H_2_ desorption of the 1-2 sample. 19](#_Toc197619720)

[Figure S22: ^11^B NMR of TBABH-AB 1-2 before and after heat treatment at 100 °C and 150 °C (solvent: DMSO-*d_6_*). 20](#_Toc197619721)

[Figure S23: FT-IR spectra of TBABH-AB 1-2 before and after heat treatment at 150 °C and 300 °C.). 20](#_Toc197619722)

[Figure S24: Picture of the TBABH-AB 1-2 sample after heat treatment to 150 °C under Ar-flow (10 K min^−1^). 21](#_Toc197619723)

[Figure S25: Isothermal H_2_ release rate as a function of time for the 1-2 mixture at different temperatures (estimated from TG data). 22](#_Toc197619724)

[Figure S26: Isothermal H_2_ release rate at 85 °C as a function of time for the 1-2 mixture. 22](#_Toc197619725)

[Figure S27: MS data of the isothermal decomposition of the 1-2 mixture at A 85°C, B 90°C, C 95°C, and D 100°C. 23](#_Toc197619726)

[Table S3: Comparison table of the H_2_ kinetics of AB mixed with ILs or DESs. 24](#_Toc197619727)

[References 24](#_Toc197619728)

# Experimental part

Ammonia Borane (AB, > 85%, TCI) and Tetrabutylammonium Borohydride (TBABH, > 97.0%, Fluorochem) were used without additional treatment.

### Preparation of the mixtures

The mixtures were prepared in an Ar-filled glovebox (Miwa, O_2_ < 0.1 ppm, H_2_O < 1 ppm) by mixing TBABH or ChCl with AB at various molar ratio with a mortar and pestle for around 5 minutes until a homogeneous liquid was obtained. The samples were stored inside the glovebox until their utilization.

### Characterization

DESs were characterised with a Fourier-transform infrared spectroscopy (FT-IR; Bruker Optics ALPHA, ATR accessory, Germany) located inside an Ar-filled glovebox, and Nuclear Magnetic Resonance spectroscopy (^1^H and ^11^B NMR; Bruker 500 MHz AVIII, Germany) with DMSO-*d*_6_ as solvent. The amorphization of the DESs were confirmed by X-ray powder diffraction (PXRD, Rigaku Miniflex, CuKα radiation, Japan) using a closed sample holder to avoid contact with air. Differential scanning calorimetry (DSC, Hitachi 7020) were performed in sealed aluminium pan prepared inside an Ar-filled glovebox. Sampled were cooled and heated at a rate of 10 °C min^-1^ under N_2_ flow. Glass transitions and melting were taken from the second heating cycle and determined from the onset value. Kissinger's analysis of the thermal decomposition was performed on a DSC (Metler Toledo HP__) with open Al-pan. Thermo-gravimetric and differential thermal analysis measurements were performed inside an Ar-filled glovebox (TG-DTA, Rigaku STA8122). The sample was placed inside an Al pan, and the measurement was carried out under Ar flow at 10 K min^−1^. In order to know the evolved gas during thermolysis, thermo-gravimetric measurement coupled with mass spectroscopy was performed (TG-MS, NETZSCH TG 209 F1 Libra, coupled with NETZSCH QMS 403 Aëolos Quadro) under an Ar or N_2_ flow at a rate of 5 K min^−1^. Complex viscosity in function of temperature and viscosity in function of shear rate were checked with a rheometer (Discovery, TA Instrument) by oscillation using an Al disk plate of 25 mm with an axial force of 1 N and an angular frequency of 10 rad s^−1^. Refractive indices with regard to the sodium D line (*n*_D_) were determined with a Reichert AR200 Portable refractometer. Densities were measured with a BELPYCNO. The *in-situ* Raman spectroscopy was measured using a temperature and environment controlled Linkam THMS 350V stage in a Raman microscope (Renishaw, inVia confocal Raman microscope) with a green light source with a wavelength of 532 nm. The sample was sealed in the cell inside an Ar-filled glovebox and cooled down to the target temperature at a rate of 10 K min^−1^ and kept 5 minutes before collecting the spectrum.

### DFT calculation

GAMESS package was used to conduct geometry optimization and vibrational analyses. We used B3LYP functional and 6-311G(d,p) basis set with diffuse functions (B3LYP/6-311++G(d,p) level).^[1,2]^ The computational time was provided by the Supercomputer System, Institute for Chemical Research, Kyoto University.

### Determination of the ideal eutectic temperature

We determine the ideal eutectic temperature for the 1-2 mixture with the Schröder-van-Laar equation^[3]^:

$\ln x_{TBABH}= \frac{\Delta_{fus}H_{TBABH}}{R\cdot T_{fus,TBABH}}\left[ 1-\frac{T_{fus,TBABH}}{T} \right]$ **Equation S1**

where $x_{TABH}$ is the mol fraction of TBABH, $\Delta_{fus}H_{TBABH}$ the enthalpy of fusion, $T_{fus,TBABH}$ the melting temperature, *R* the gas constant, and *T* the temperature.

If we solve for *T*, the equation becomes:

$T=\frac{\Delta_{fus}H_{TBABH}\cdot T_{fus,TBABH}}{\Delta_{fus}H_{TBABH}-R\cdot T_{fus,TBABH}\cdot\ln x_{TBABH}}$ **Equation S2**

# Results


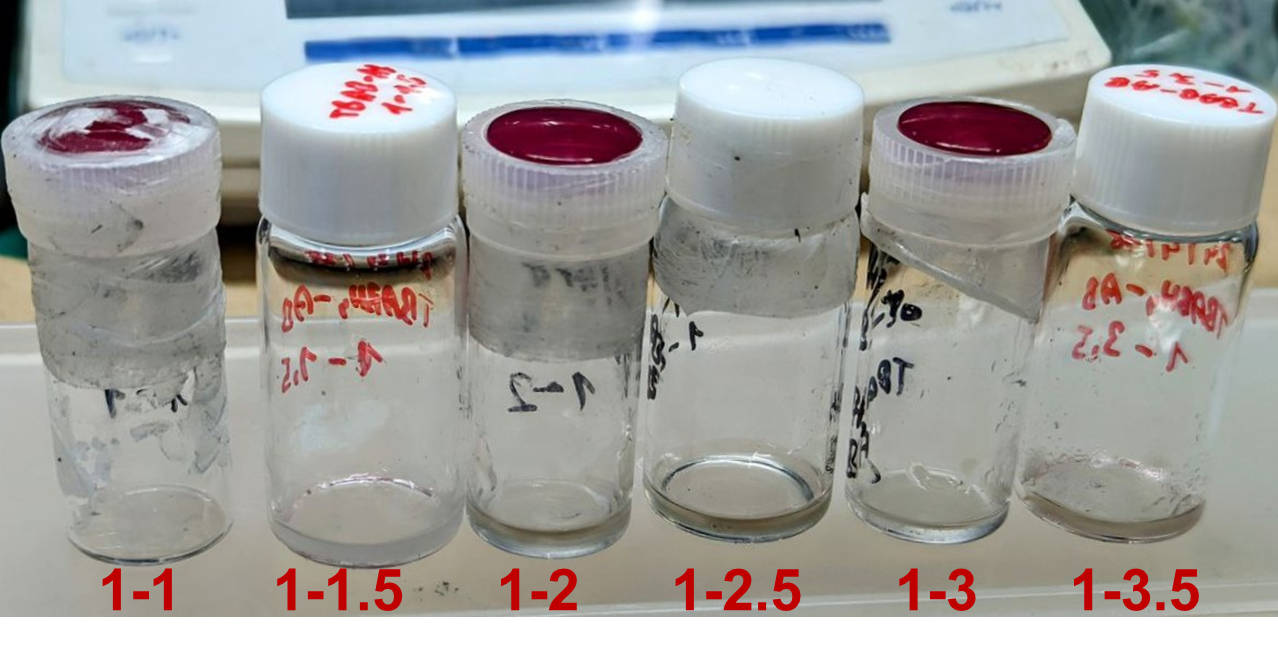


### Figure S1: Pictures of the as-prepared DESs made from TBABH and AB with different molar ratio (TBABH-AB).


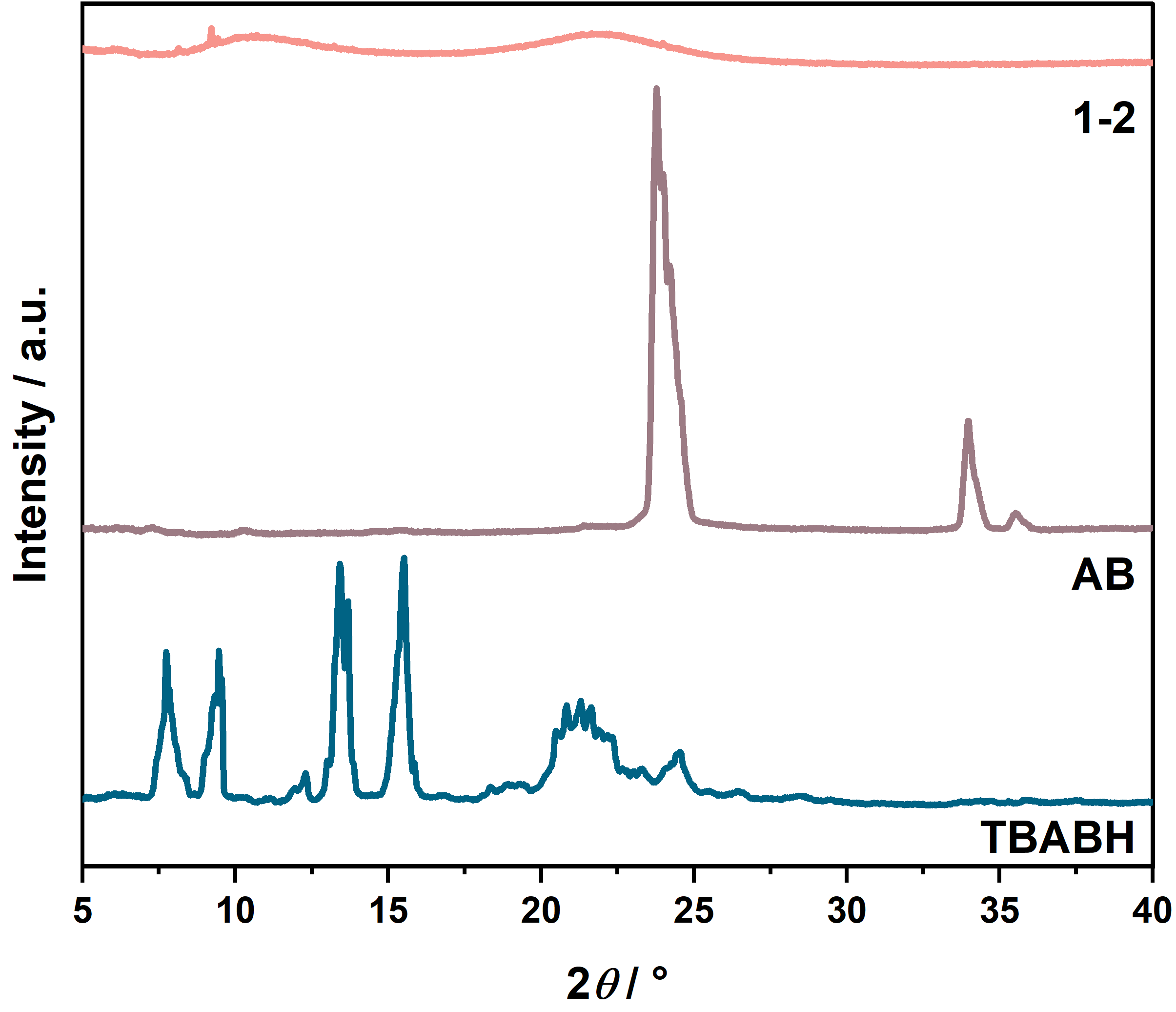


### Figure S2: PXRD pattern of TBABH, AB, and TBABH-AB 1-2.

### Figure S3: Theoretical hydrogen content of the AB and TBABH mixtures forming liquids based on the mole equivalent of AB.


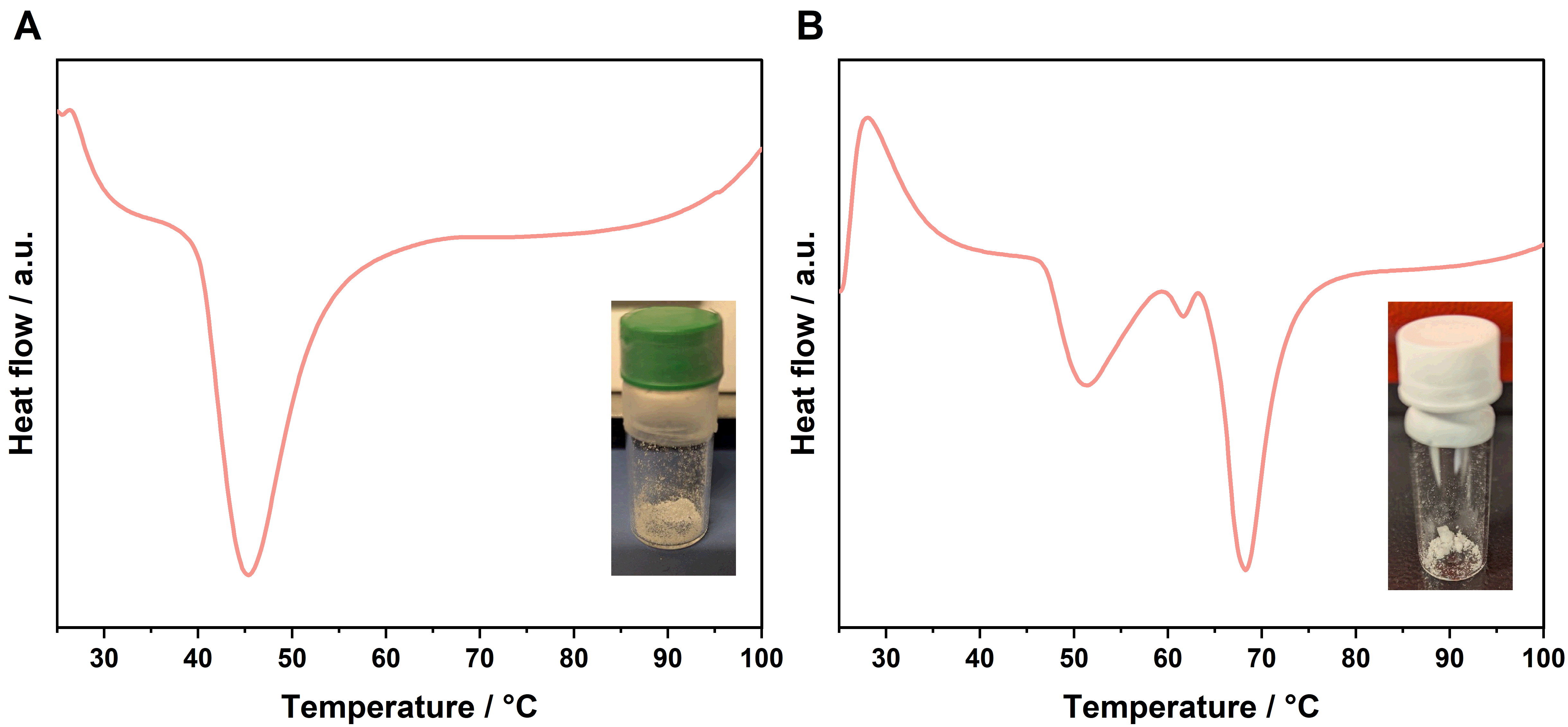


### Figure S4: DSC profile of A TMABH-AB 1-2 and B TEABH-AB 1-2 heated to 100 °C. The endothermic peaks observed are due to partial melting or phase change, but it was not possible to produce a clear liquid at temperature below the decomposition of AB.


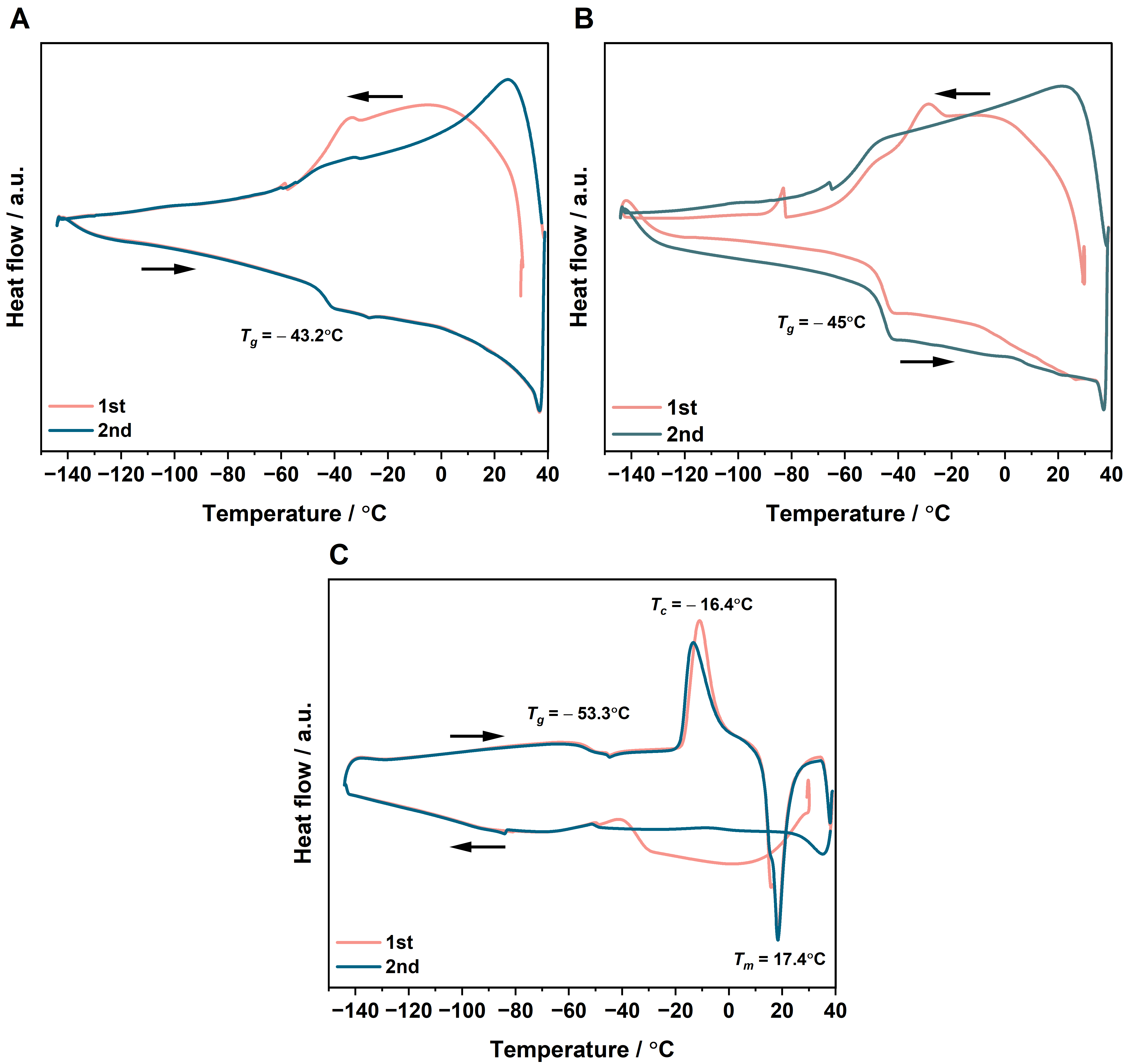


### **Figure S5:** First and second cycles DSC profile of A TBABH-AB 1-1.5, B TBABH-AB 1-2.5, and C TBABH-AB 1-3.5 cooled down up to -140 °C at 10 K min^−1^.


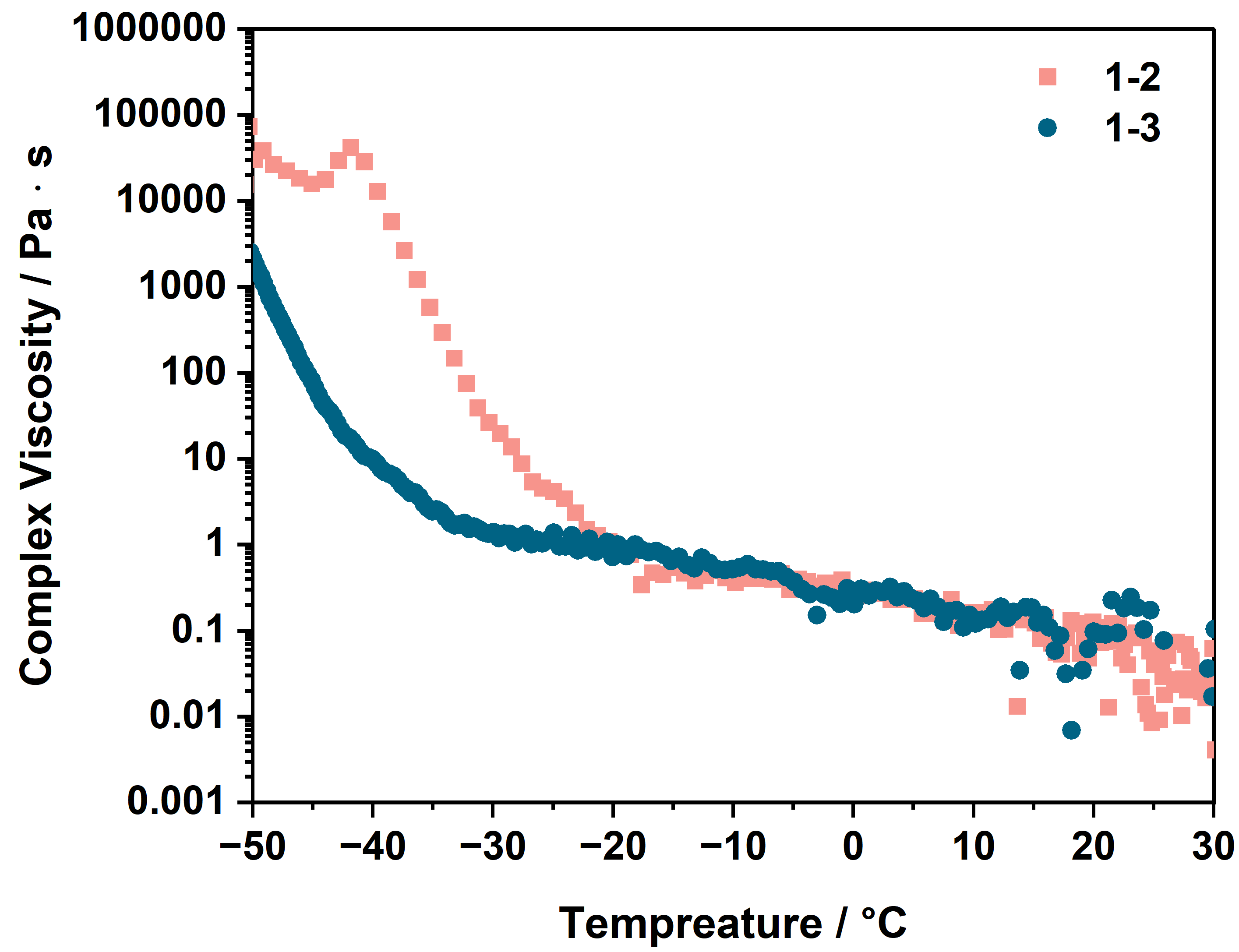


### Figure S6: Complex viscosity of the 1-2 and 1-3 DESs as a function of temperature. Cooling rate 5 K min^−1^, angular frequency 10 rad s^−1^,


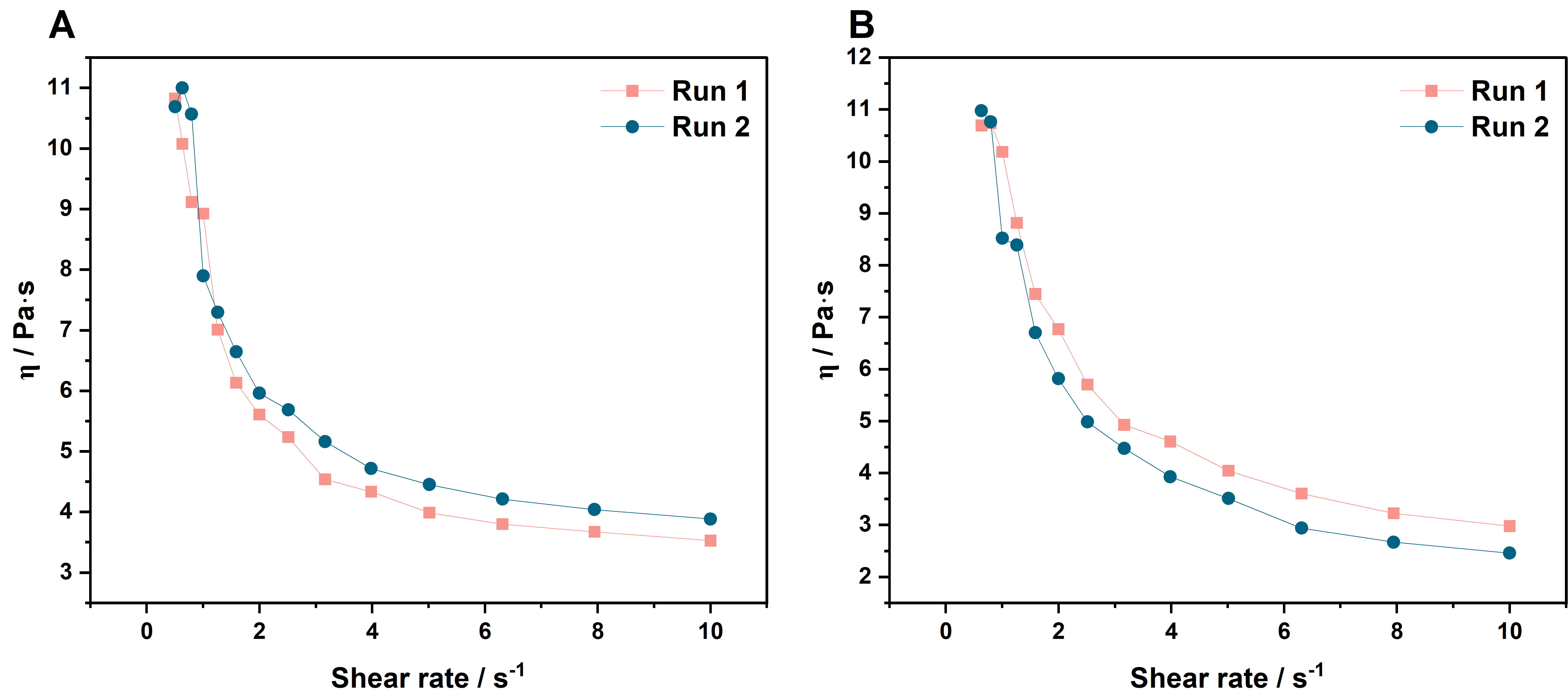


### **Figure S7:** Viscosity as a function of the shear rate for A 1-2 and B 1-3 over two runs.


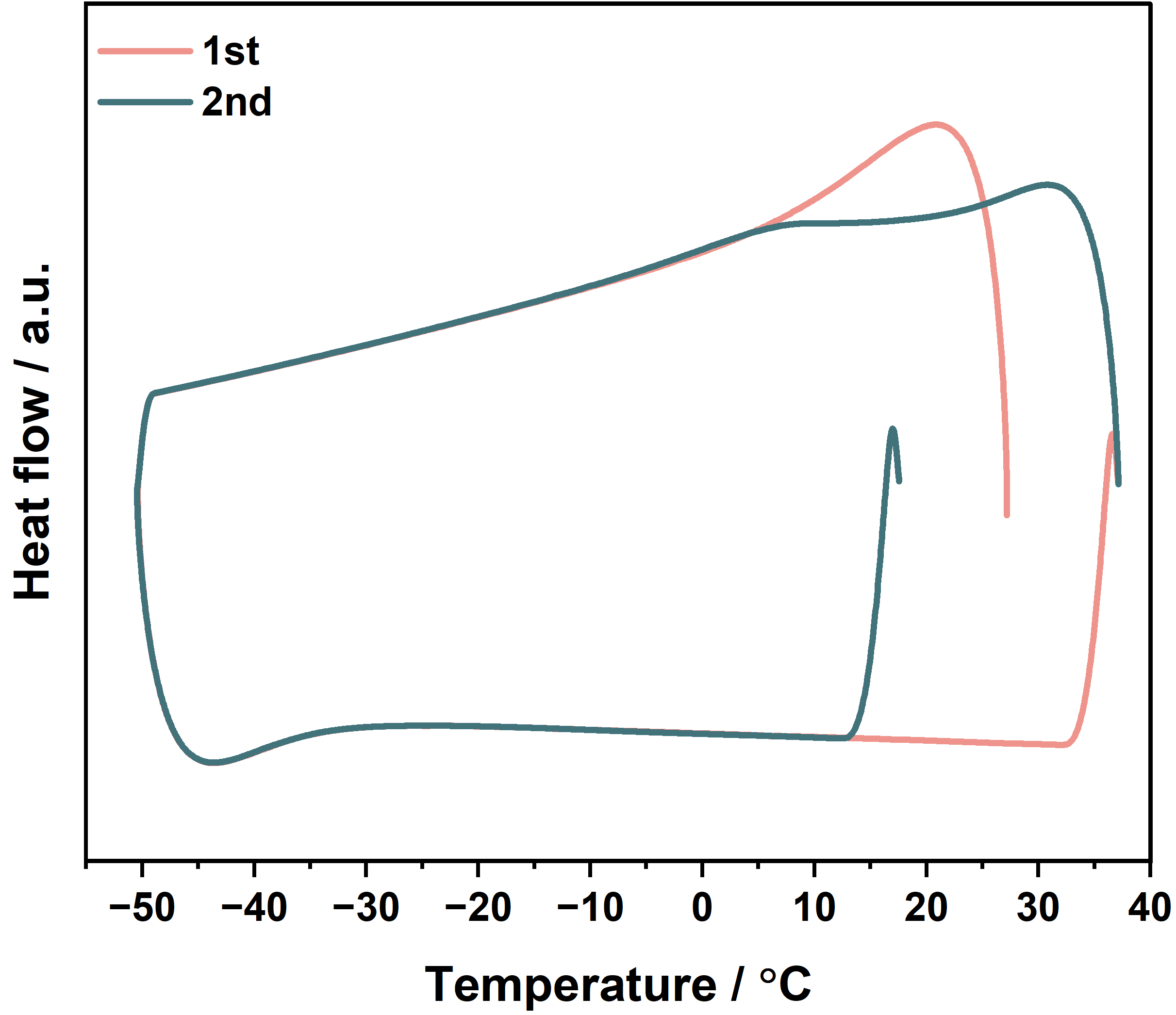


### **Figure S8:** First and second cycles DSC profile of TBABH-AB 1-3 cooled down up to -50 °C at 10 K min^−1^.


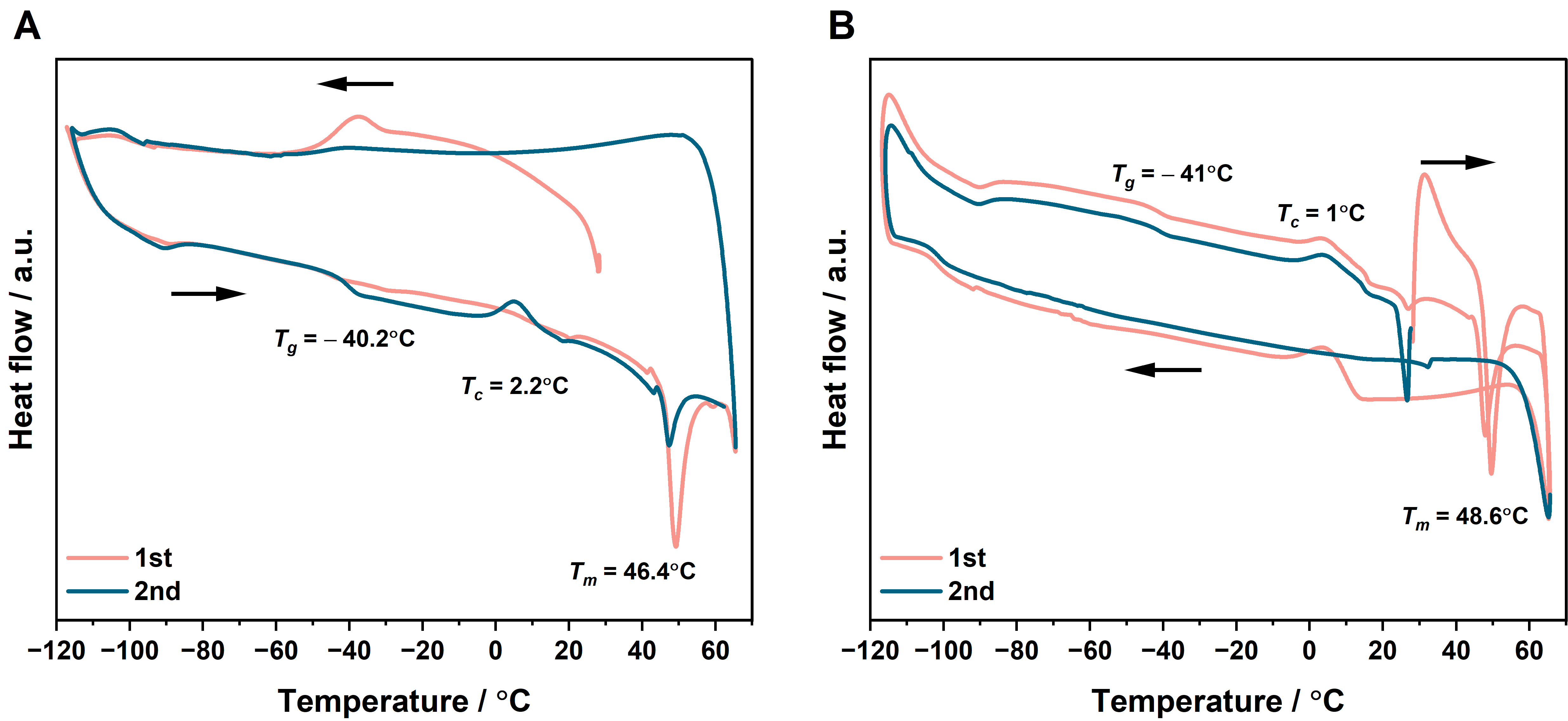


### **Figure S9:** First and second cycles DSC profile of A TBABH-AB 1-0.75 and B TBABH-AB 2-1 cooled down to -140 °C and heated up to 65 °C at 10 K min^−1^.


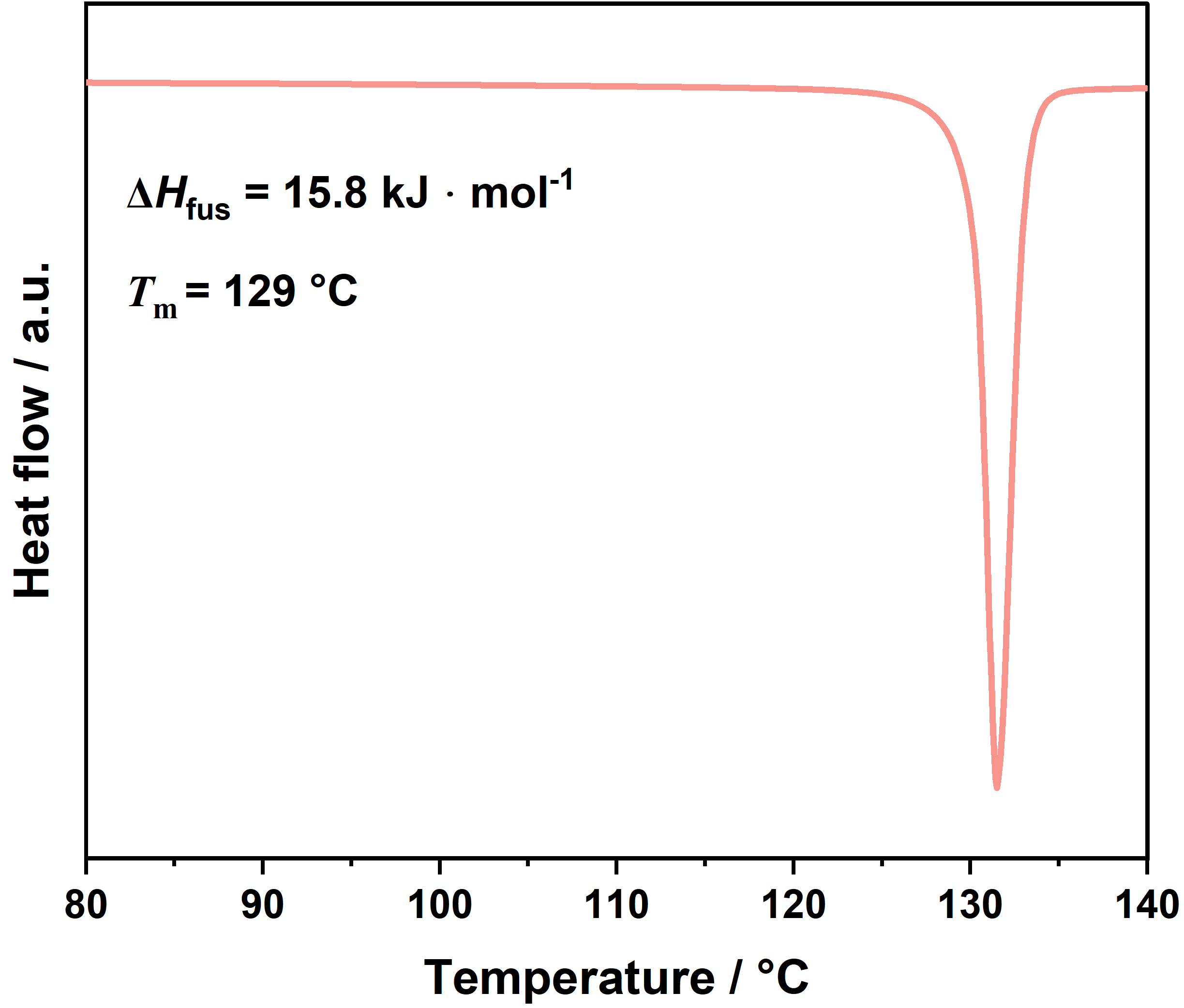


### **Figure S10:** DSC curve of TBABH melting to determine the melting enthalpy.


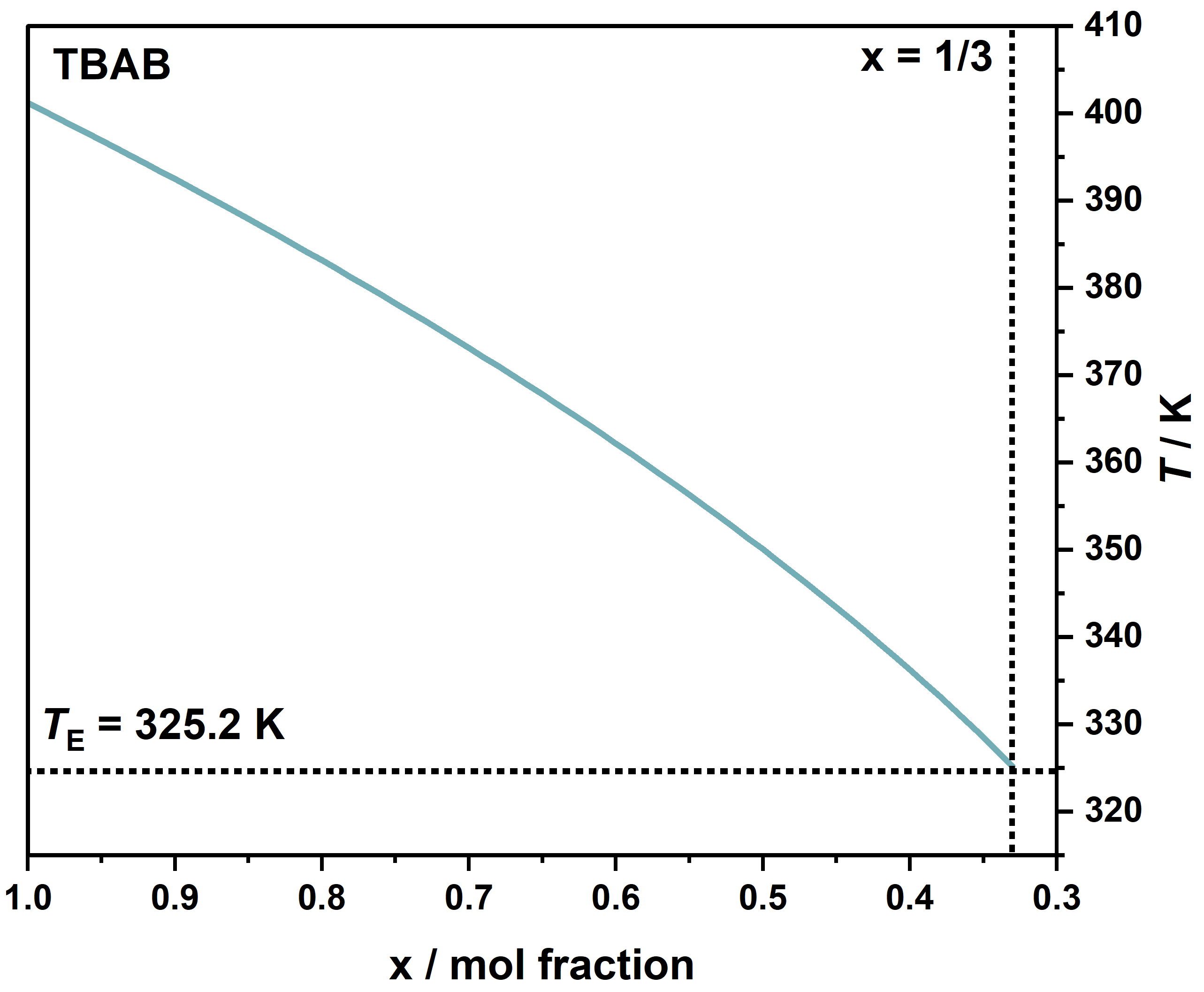


### **Figure S11:** Estimation of the theoretical eutectic temperature from the Schröder-van-Laar equation.

Table S1: Physicochemical properties of the 1-2 and 1-3 DES.

|  | 1-2 | 1-3 |
| --- | --- | --- |
| Refractive index *n*_D_ (25°C) | 1.4771 | 1.4753 |
| Viscosity at a shear rate of 10 s^−1^ (25°C) | 3.71 ± 0.25 Pa·s | 2.72 ± 0.37 Pa·s |
| Density (25°C) | 0.766 g·cm^−3^ | 0.747 g·cm^−3^ |


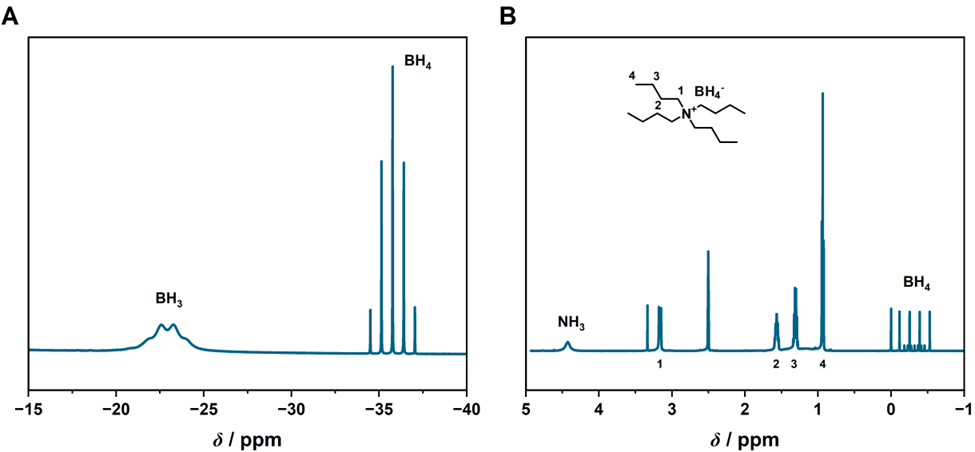


### **Figure S12: A** ^11^B and B ^1^H NMR of TBABH-AB 1-2 (solvent: DMSO-*d_6_*).


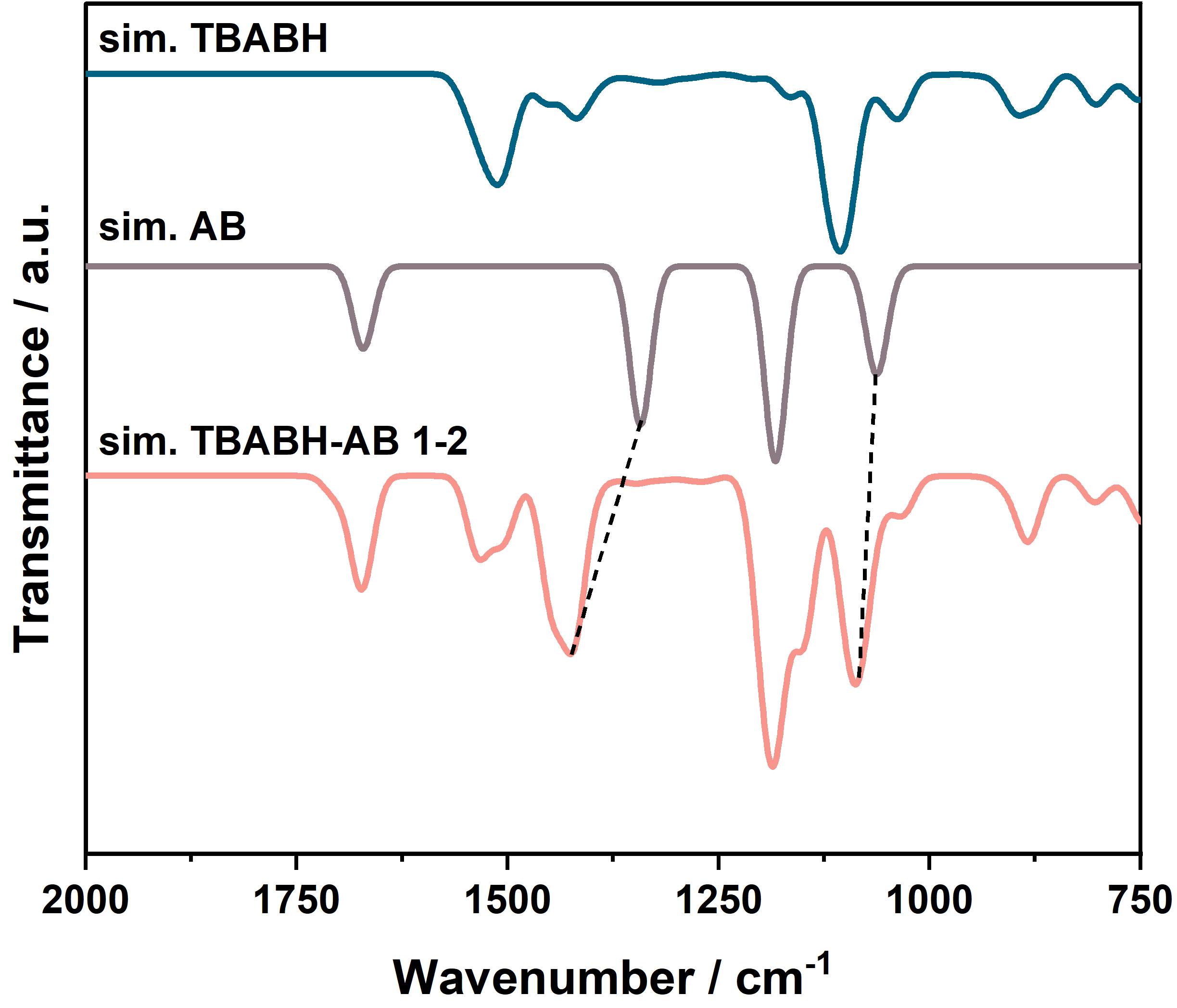


### Figure S13: Simulated FT-IR spectra of TBABH, AB, and TBABH-AB 1-2.


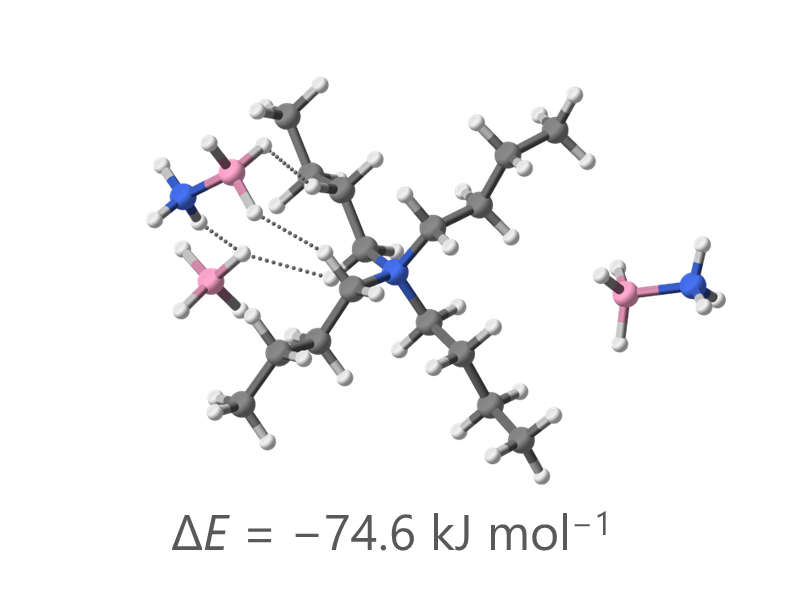


### Figure S14: Gas phase optimized molecular structure of 1-2 TBABH-AB DES with only one AB interacting with BH_4_^−^ using B3LYP/6-311++G(d,p).


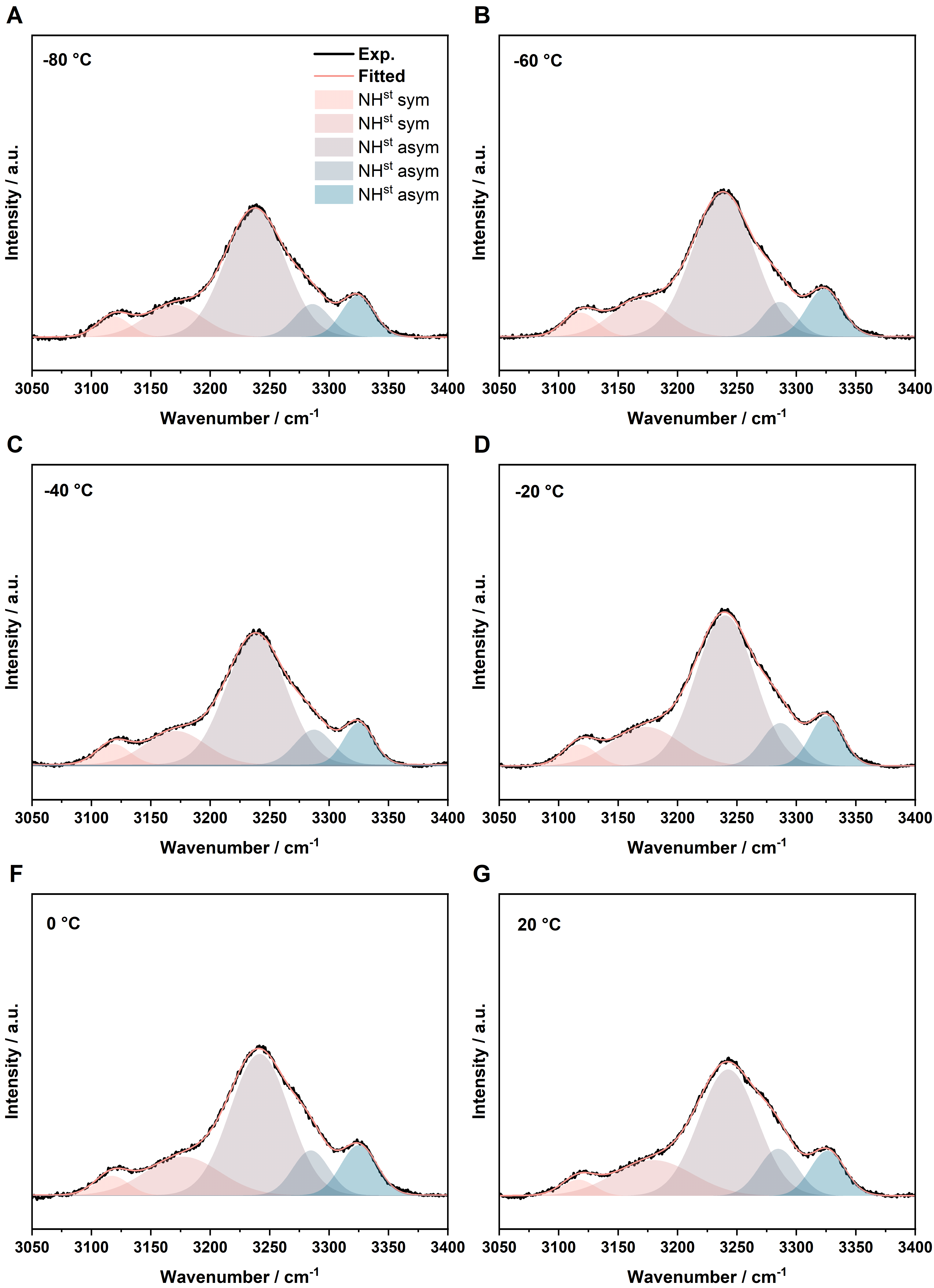


### Figure S15: *In-*situ Raman spectra of the NH^st^ region and their deconvolution for TBABH-AB 1-2 DES at A −80 °C, B −60 °C, C −40 °C, D −20 °C, E 0 °C, and F 20 °C.

### Table S2: Calculated Raman peak position in the NH_3_ region based on the DFT model.

| Vibration mode | Position / cm^−1^ |
| --- | --- |
| Sym | 3246.20 |
| Sym | 3259.84 |
| Asym | 3504.59 |
| Asym | 3507.10 |
| Asym | 3570.15 |
| Asym | 3570.57 |


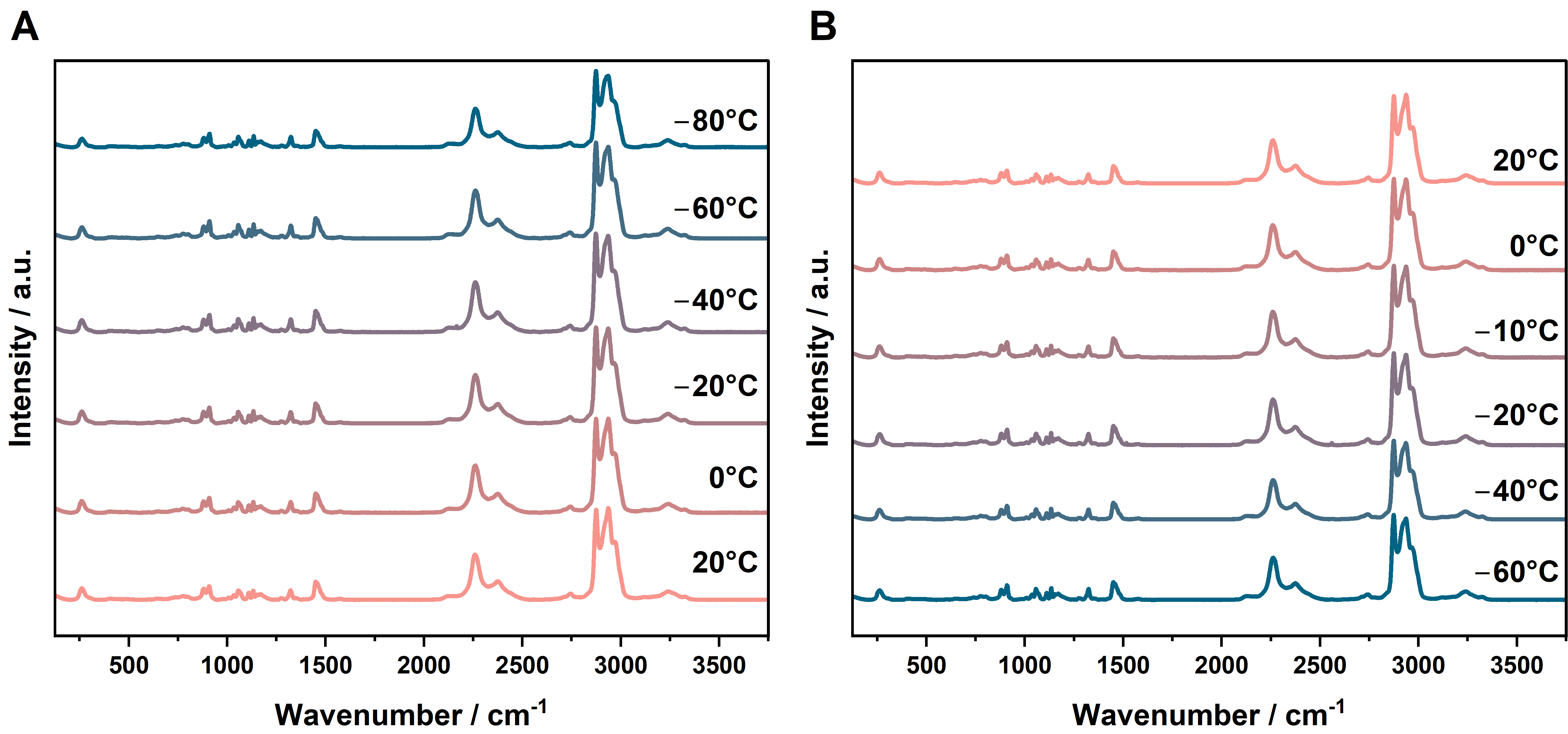


### Figure S16: *In-*situ Raman spectra of TBABH-AB 1-2 DES during A cooling to −80 °C and B heating back to 20 °C. Cooling/heating rate: 10 K min^−1^.


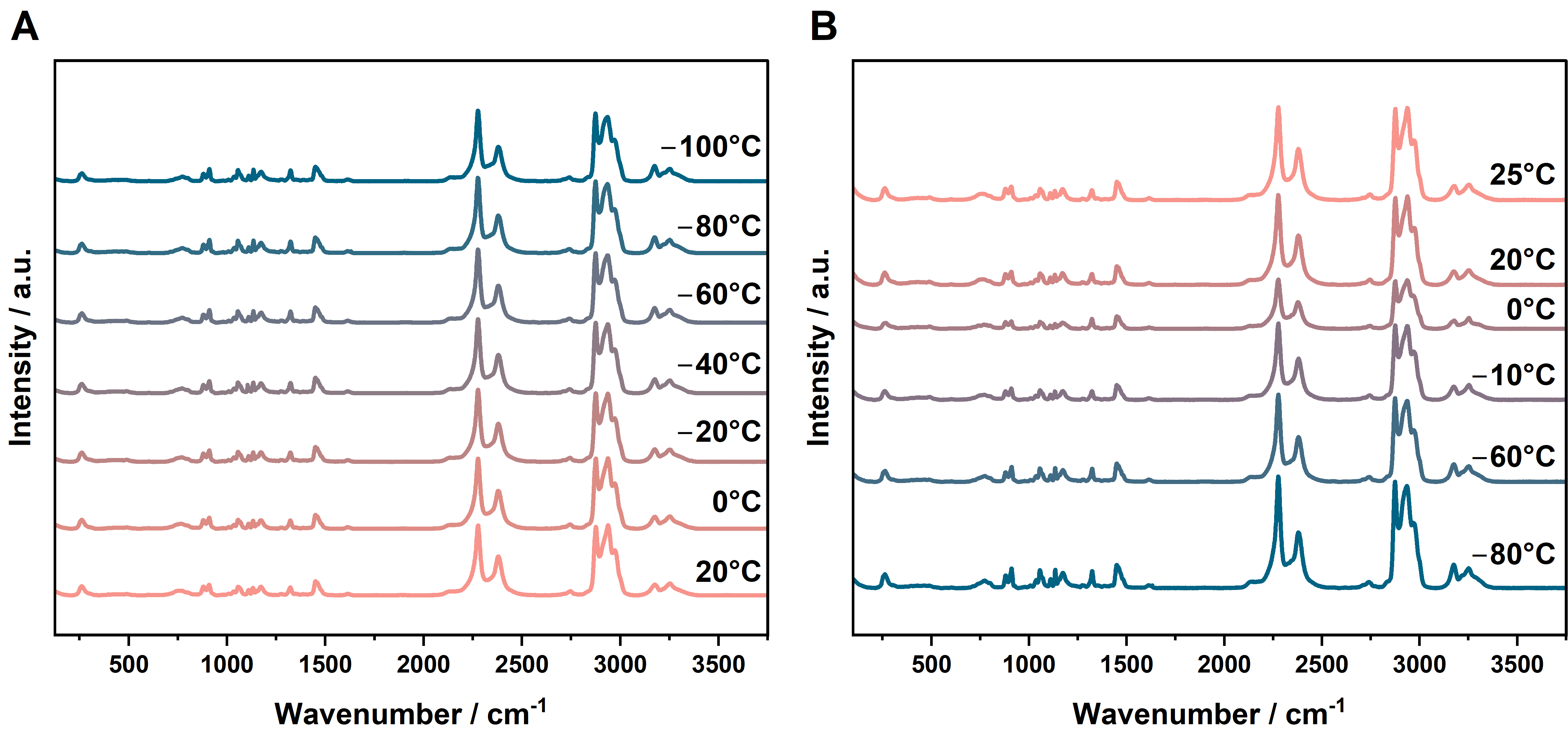


### Figure S17: *In-*situ Raman spectra of TBABH-AB 1-3 DES during A cooling to −100 °C and B heating back to 25 °C. Cooling/heating rate: 10 K min^−1^.


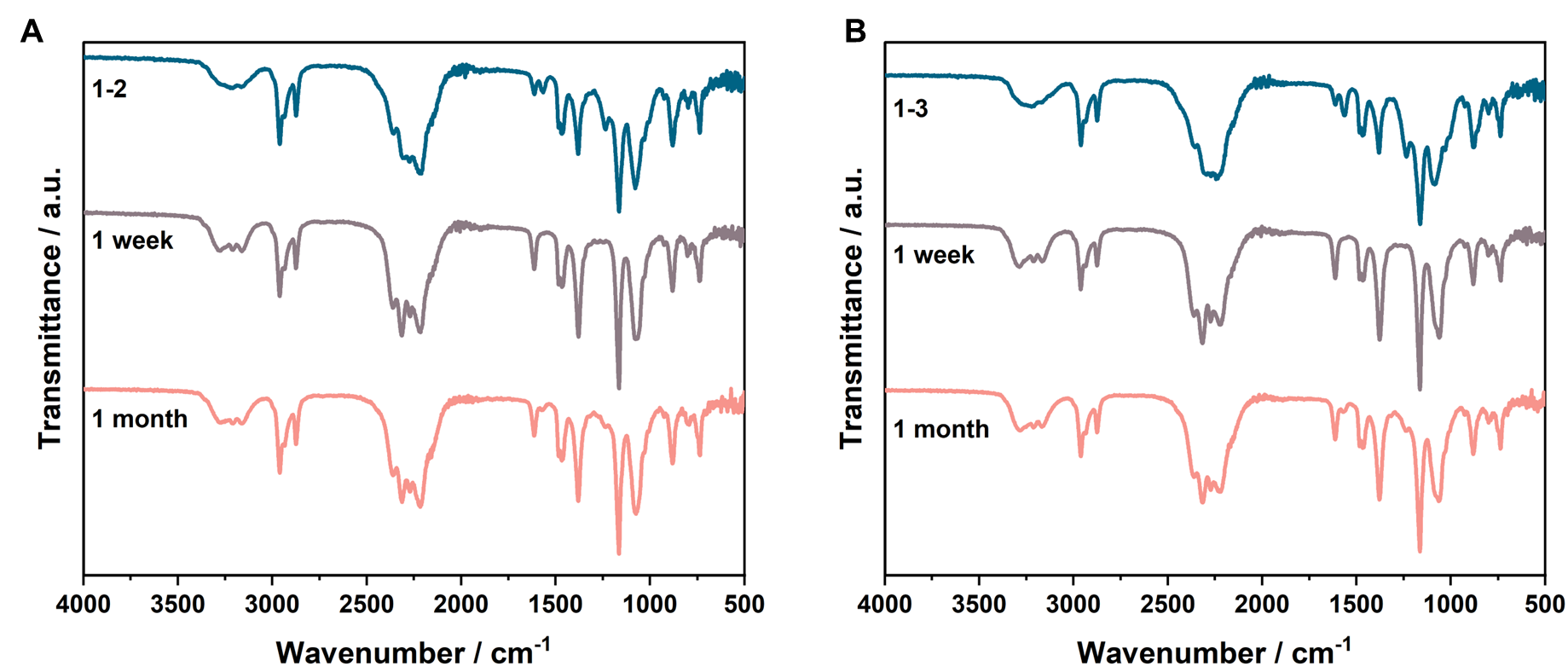


### Figure S18: Evolution of FT-IR spectra over time for A TBABH-AB 1-2 and B TBABH-AB 1-3.


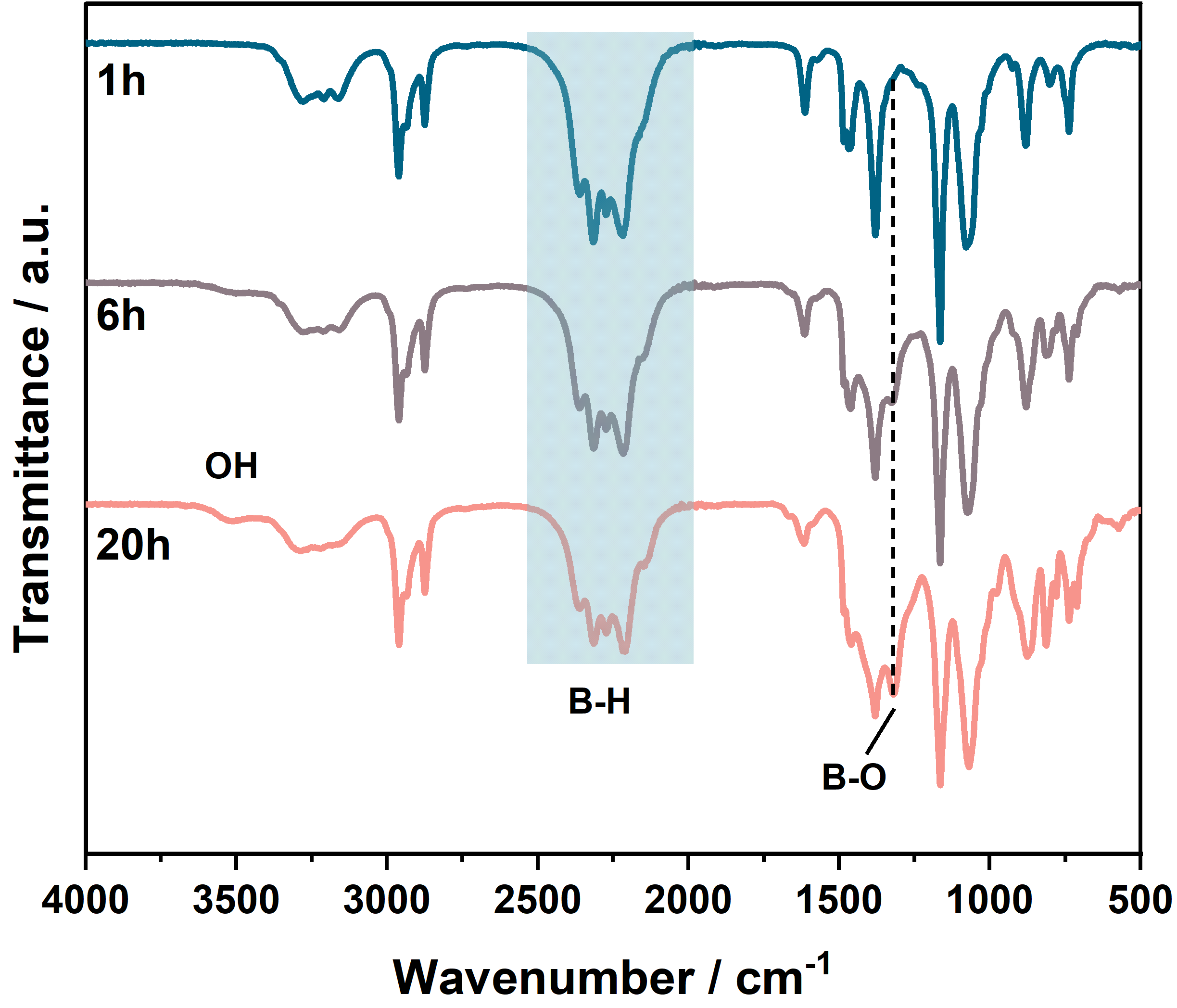


### Figure S19: Evolution of FT-IR spectra over time for TBABH-AB 1-2 exposed to air (20 °C, RH 30%).


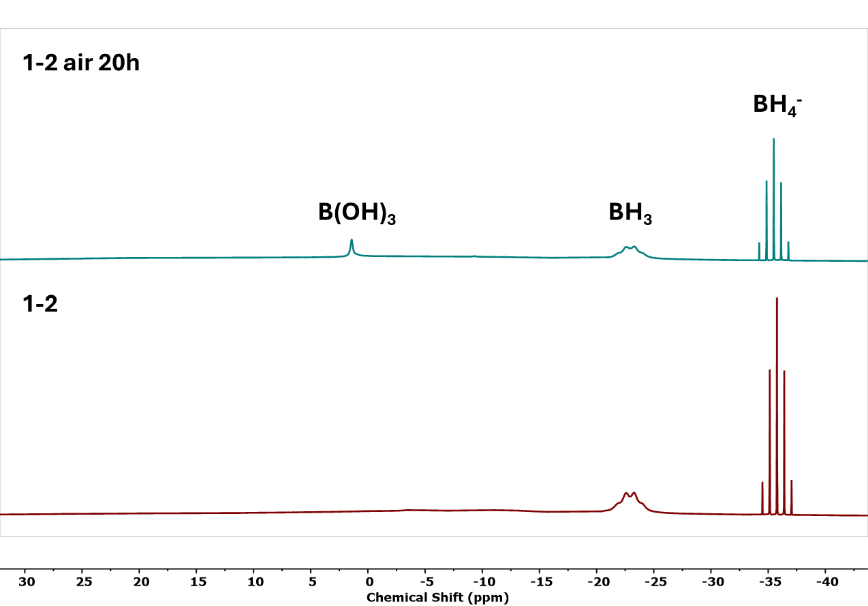


### Figure S20: ^11^B NMR of the 1-2 DES before and after 20h air exposure (solvent: DMSO-*d_6_*).


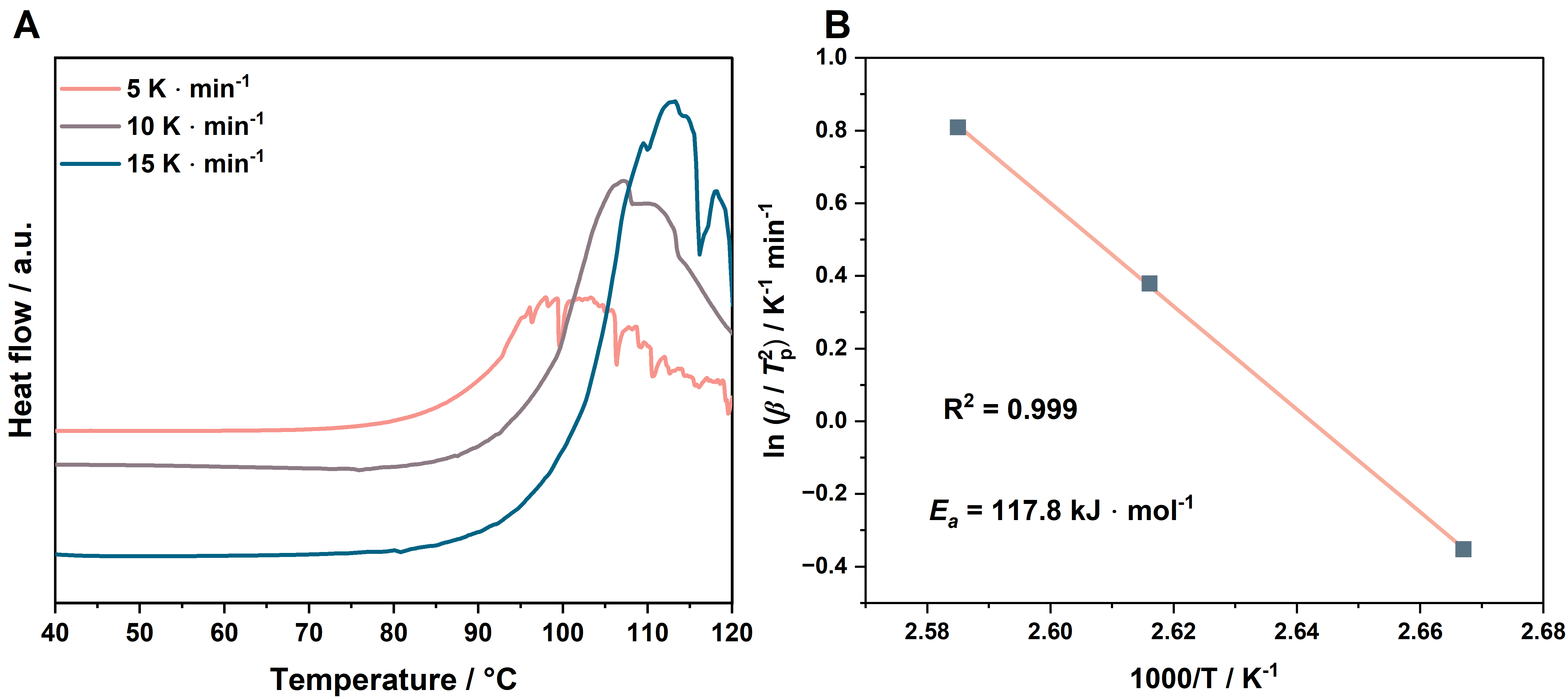


### Figure S21: Kissinger analysis on the H_2_ desorption of the 1-2 sample.


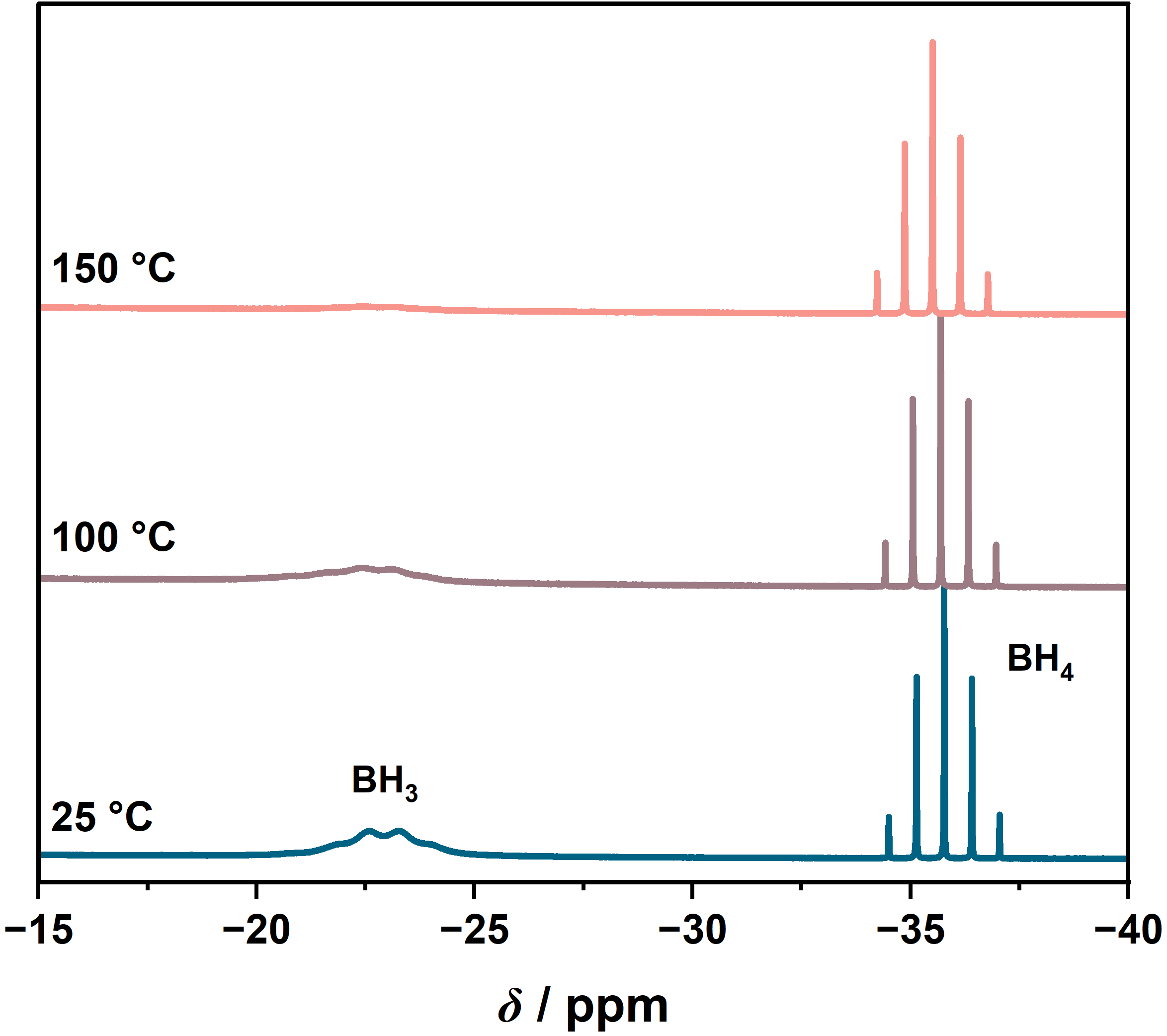


### Figure S22: ^11^B NMR of TBABH-AB 1-2 before and after heat treatment at 100 °C and 150 °C (solvent: DMSO-*d_6_*).


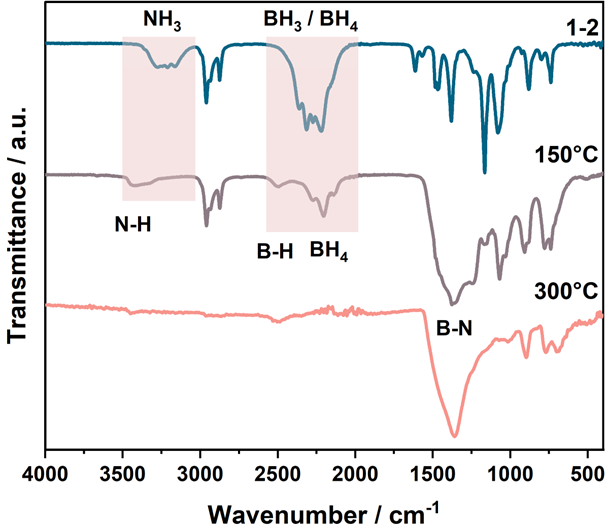


### Figure S23: FT-IR spectra of TBABH-AB 1-2 before and after heat treatment at 150 °C and 300 °C.).


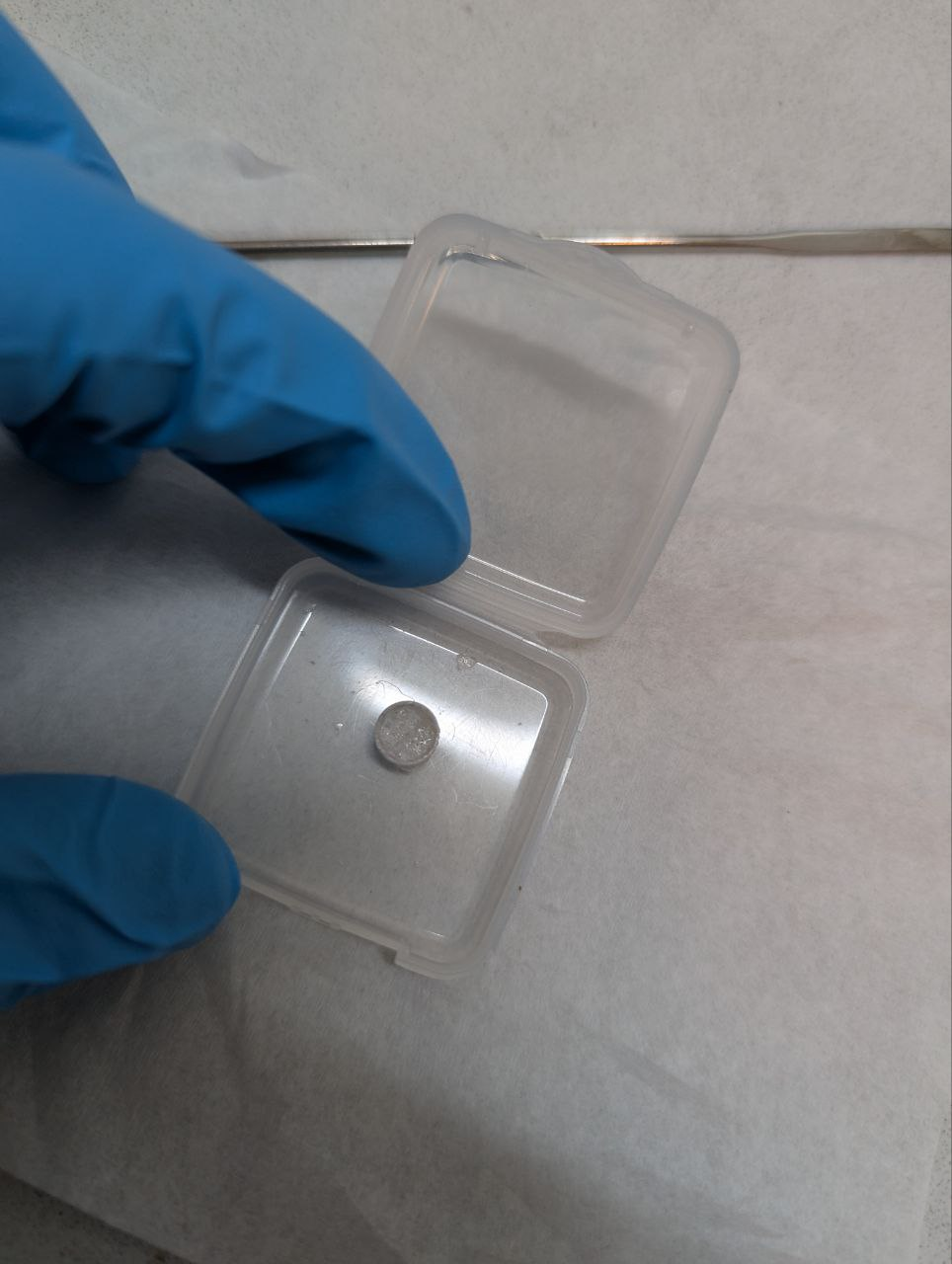


### Figure S24: Picture of the TBABH-AB 1-2 sample after heat treatment to 150 °C under Ar-flow (10 K min^−1^).


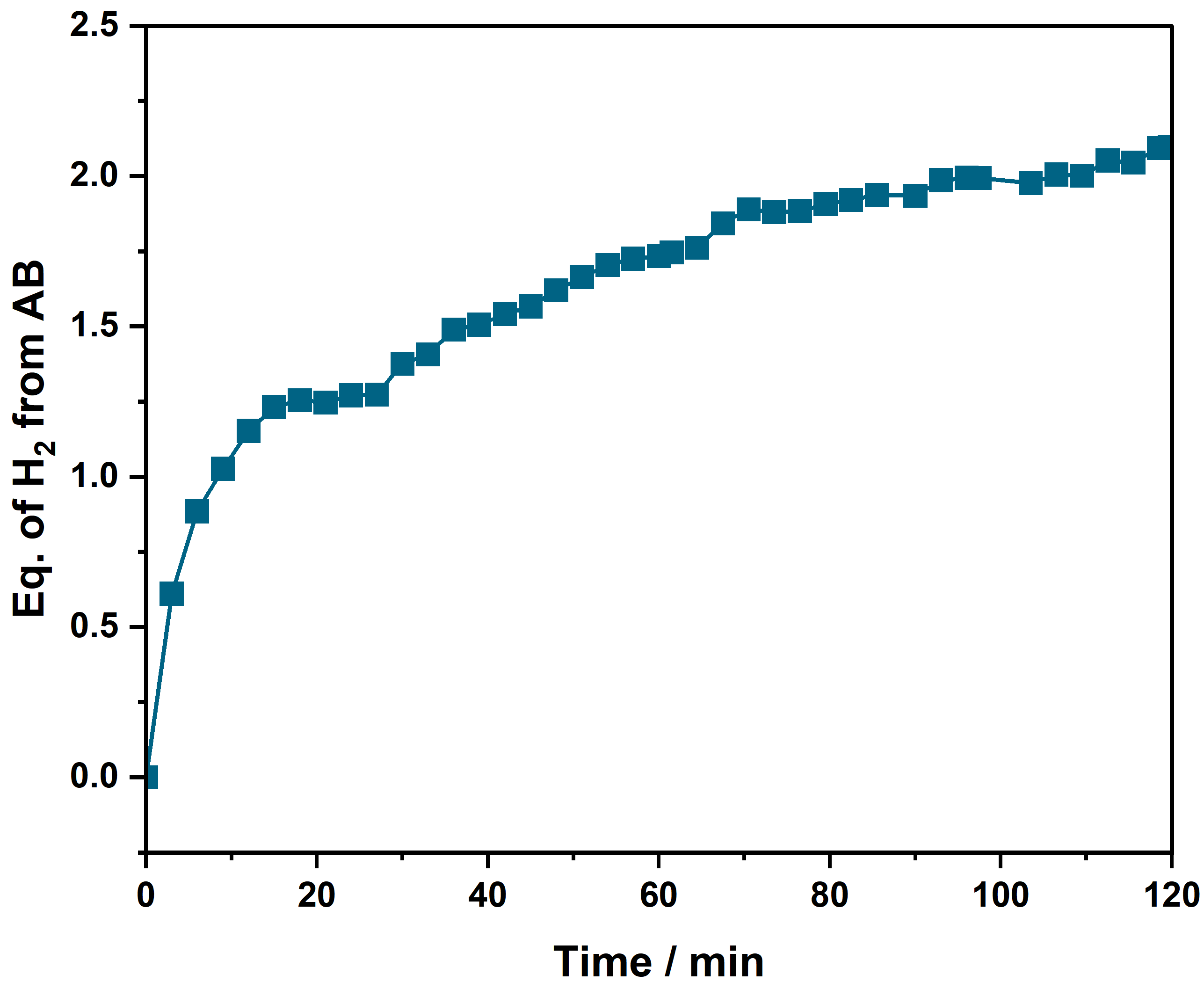

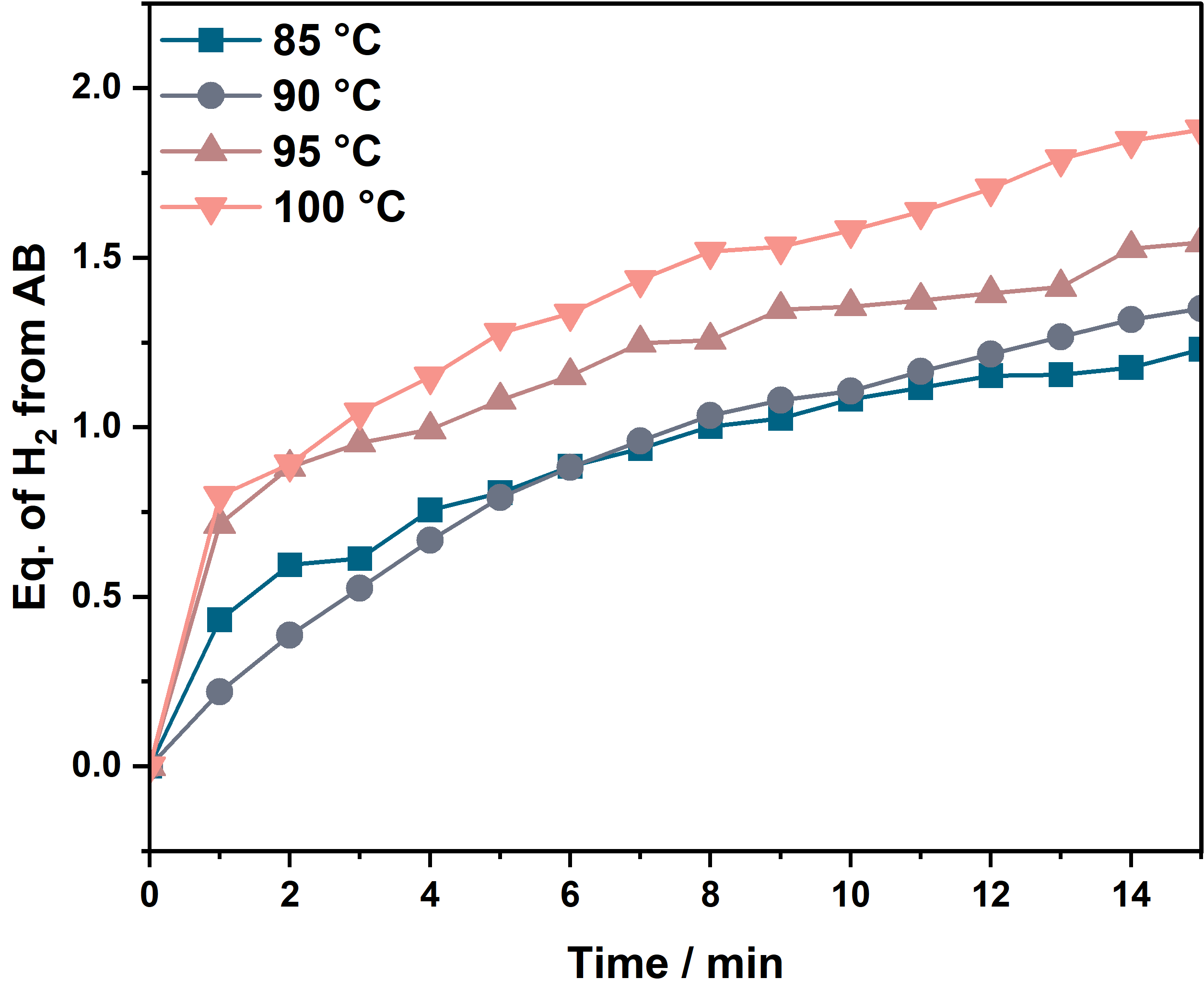


### Figure S25: Isothermal H_2_ release rate as a function of time for the 1-2 mixture at different temperatures (estimated from TG data).

### Figure S26: Isothermal H_2_ release rate at 85 °C as a function of time for the 1-2 mixture.


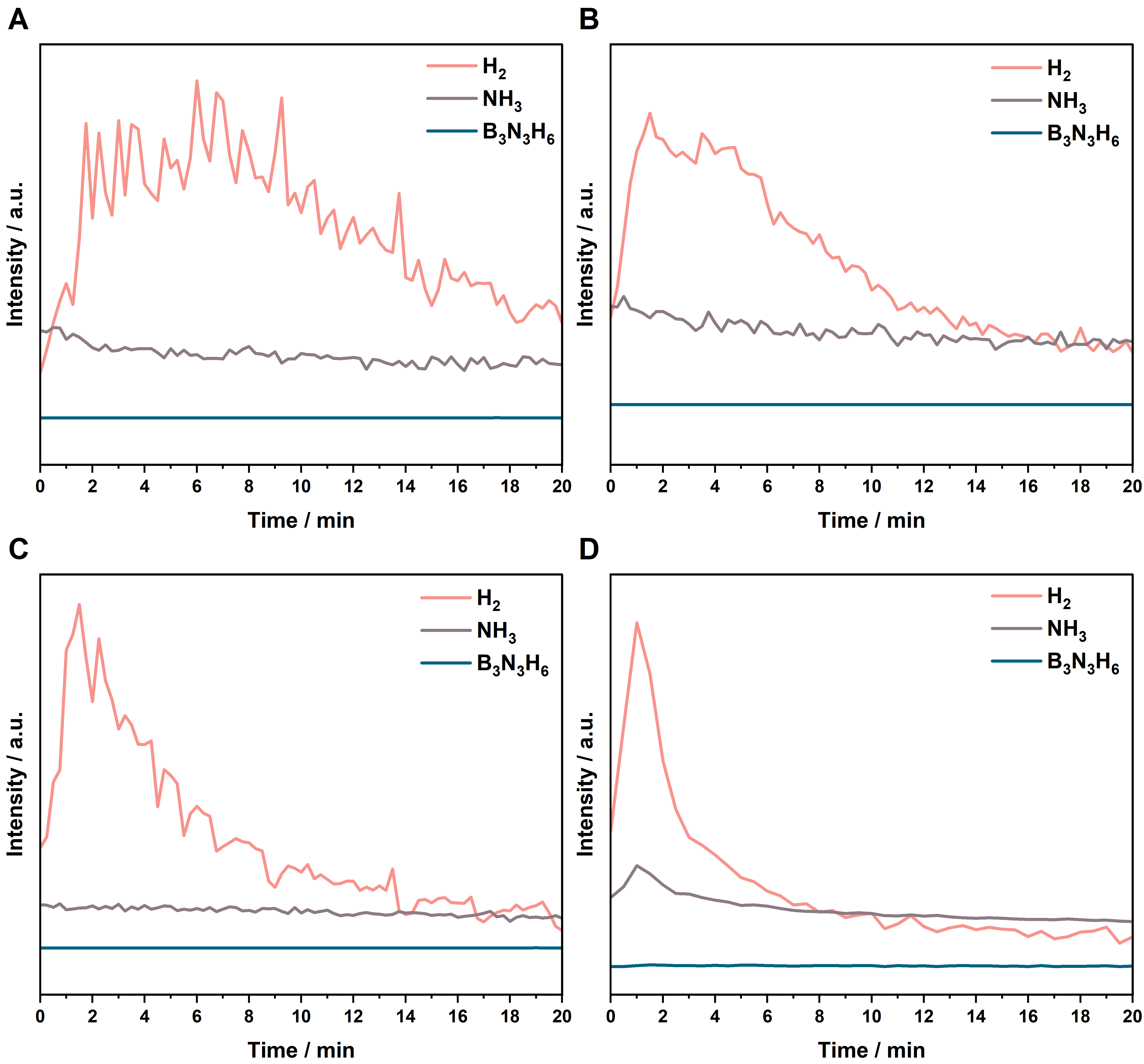


### Figure S27: MS data of the isothermal decomposition of the 1-2 mixture at A 85°C, B 90°C, C 95°C, and D 100°C.

### Table S3: Comparison table of the H_2_ kinetics of AB mixed with ILs or DESs.

| System | Available H_2_ / wt% | *T*_d_ / °C | Rate for 1 eq. of H_2_ | Ref. |
| --- | --- | --- | --- | --- |
| AB/bmimCl | 9.8 | / | 1 h @ 95°C  6 h @ 85°C | [4] |
| AB/bmimCl | 9.8 | / | 67 min @ 85°C | [5] |
| AB-bmimCl | 4.63 | 110 | / | [6] |
| AB/[TBMA][CH_3_CO_3_] | 0.5 | / | 800 min @ 95°C | [7] |
| AB/DES ([bmim][MeSO_3_]:[Im] 1:2) | 0.5 | / | 75 min @ 105°C | [8] |
| TBABH-AB 1-2 | **5.1** | **60** | **8 min @ 85°C** | **This work** |

# References

[1] S. Grimme, J. Antony, S. Ehrlich, H. Krieg, *J. Chem. Phys.* **2010**, *132*, 154104.

[2] S. Grimme, S. Ehrlich, L. Goerigk, *J. Comput. Chem.* **2011**, *32*, 1456.

[3] M. Prencipe, P. P. Mazzeo, A. Bacchi, *RSC Mechanochemistry* **2025**, *2*, 61.

[4] M. E. Bluhm, M. G. Bradley, R. Butterick, U. Kusari, L. G. Sneddon, *J. Am. Chem. Soc.* **2006**, *128*, 7748.

[5] D. W. Himmelberger, L. R. Alden, M. E. Bluhm, L. G. Sneddon, *Inorg. Chem.* **2009**, *48*, 9883.

[6] T. Nakagawa, A. K. Burrell, R. E. D. Sesto, M. T. Janicke, A. L. Nekimken, G. M. Purdy, B. Paik, R.-Q. Zhong, T. A. Semelsberger, B. L. Davis, *RSC Adv.* **2014**, *4*, 21681.

[7] D. K. Mishra, B. Banerjee, G. Pugazhenthi, T. Banerjee, *Ind. Eng. Chem. Res.* **2021**, *60*, 9764.

[8] D. K. Mishra, G. Pugazhenthi, T. Banerjee, *Int. J. Hydrog. Energy* **2021**, *46*, 15773.
